# Spatially aligned graph transfer learning for characterizing spatial regulatory heterogeneity

**DOI:** 10.1093/bib/bbaf021

**Published:** 2025-01-22

**Authors:** Wendong Huang, Yaofeng Hu, Lequn Wang, Guangsheng Wu, Chuanchao Zhang, Qianqian Shi

**Affiliations:** Hubei Key Laboratory of Agricultural Bioinformatics, College of Informatics, Huazhong Agricultural University, Wuhan 430070, China; Hubei Engineering Technology Research Center of Agricultural Big Data, College of Informatics, Huazhong Agricultural University, Wuhan 430070, China; Key Laboratory of Systems Health Science of Zhejiang Province, School of Life Science, Hangzhou Institute for Advanced Study, University of Chinese Academy of Sciences, Hangzhou 310024, China; State Key Laboratory of Cell Biology, Shanghai Institute of Biochemistry and Cell Biology, Center for Excellence in Molecular Cell Science, Chinese Academy of Sciences, Shanghai 200031, China; School of Mathematics and Computer Science, Xinyu University, Xinyu 338004, Jiangxi, China; Key Laboratory of Systems Health Science of Zhejiang Province, School of Life Science, Hangzhou Institute for Advanced Study, University of Chinese Academy of Sciences, Hangzhou 310024, China; Hubei Key Laboratory of Agricultural Bioinformatics, College of Informatics, Huazhong Agricultural University, Wuhan 430070, China; Hubei Engineering Technology Research Center of Agricultural Big Data, College of Informatics, Huazhong Agricultural University, Wuhan 430070, China

**Keywords:** spatially resolved transcriptomics, spatial regulatory network inference, graph transformers, cross-dimensional transfer learning

## Abstract

Spatially resolved transcriptomics (SRT) technologies facilitate the exploration of cell fates or states within tissue microenvironments. Despite these advances, the field has not adequately addressed the regulatory heterogeneity influenced by microenvironmental factors. Here, we propose a novel Spatially Aligned Graph Transfer Learning (SpaGTL), pretrained on a large-scale multi-modal SRT data of about 100 million cells/spots to enable inference of context-specific spatial gene regulatory networks across multiple scales in data-limited settings. As a novel cross-dimensional transfer learning architecture, SpaGTL aligns spatial graph representations across gene-level graph transformers and cell/spot-level manifold-dominated variational autoencoder. This alignment facilitates the exploration of microenvironmental variations in cell types and functional domains from a molecular regulatory perspective, all within a self-supervised framework. We verified SpaGTL’s precision, robustness, and speed over existing state-of-the-art algorithms and show SpaGTL’s potential that facilitates the discovery of novel regulatory programs that exhibit strong associations with tissue functional regions and cell types. Importantly, SpaGTL could be extended to process multi-slice SRT data and map molecular regulatory landscape associated with three-dimensional spatial-temporal changes during development.

## Introduction

Studying complex tissues extends beyond the molecular profiling of numerous cells; it necessitates an understanding of how the spatial context influences cellular states or functions. The transcriptional state of a cell can be modulated by a gene regulatory network (GRN), which represents a collection of regulatory interactions between transcription factors (TFs) and their downstream target genes. GRNs are often wired as intracellular signaling, diverse across cell types or developmental stages, and can also be impacted by extracellular signals, e.g., from neighboring cells within the spatial context via cell–cell communications [1]. Mapping GRNs is essential for unraveling how cellular identities are established, maintained, and altered in response to microenvironmental influences at the molecular level. The recent breakthroughs in spatially resolved transcriptomics (SRT) technologies, which enable transcriptomic profiling while preserving locational information [2–5], have opened unprecedented opportunities for capturing contextual transcriptional states. Consequently, these advances facilitate the reverse engineering of GRNs that are essential in governing diverse cellular fates influenced by microenvironmental variations.

Recent computational methods developed for SRT studies have led to new biological insights in resolving complex tissue architecture [6–11], but those for inference of GRNs underlying spatial cell states are still limited. Current methods commonly used for inferring GRNs of cell states or types primarily leverage single-cell RNA sequencing (scRNA-seq) data to identify significant co-expression patterns of TF and target genes. These scRNA-seq-based GRN (scGRN) inference algorithms often employ models that excel at capturing local linear or non-linear gene relationships among cells. For instance, Genie3 (and its successor GRNboost2) utilizes random forest regression models to train for each target against all TFs for identifying the regulatory effects on genes [12]. PIDC employs partial information decomposition to identify complex gene relationships by decomposing the mutual information between genes into different components [13]. DeepSEM constructs a neural network architecture that mirrors the structure of GRNs to learn the gene regulatory relationships by training the network weights in an end-to-end manner [14]. SINCERITIES applies regularized linear regression and partial correlation analysis to reconstruct GRNs based on temporal gene expression fluctuations, assuming that the expression change of a target gene linearly depends on the expression changes of TFs after a time delay [15]. Despite these, the recent proposed scGRN inference methods have incorporated TF perturbation analysis [16], fine-tuning on the large-volume foundation [17], or refinement by integrating TF binding sequence information (e.g., scATAC-seq data) [18, 19] to improve the inference accuracy or cell-identity specificity of regulatory networks.

However, these approaches encounter new challenges when applied to SRT studies owing to the characteristics of SRT data [20, 21]. The lack of spatial information in the models critically neglects the influence of spatial context on TF-gene associations. This oversight can result in failure to identify regulatory heterogeneity for the same cell type or state across different cellular niches despite their distinct functions within the tissue. Besides, harnessing spatially proximal co-expression patterns across putative cells or spots becomes essential for predicting statistically significant gene regulations, given the increased sparsity and reduced gene capturing efficiency observed in SRT data compared to scRNA-seq data [22]. The current GRN inference approaches proposed for SRT data, e.g., Bayesian-based SpaceX [20] and graph-based Hotspot [21], have incorporated the co-expression similarity in spatial proximity in addition to that within cell types or states. However, they often rely on prior knowledge as input, e.g., cell cluster or data annotation, to guide the network inference. However, precise cell segmentation remains challenging for the data generated from mainstream SRT technologies [6–11], undoubtedly raising higher demands for computational methods with less restricted to data with accompanying labels. Moreover, these methods can only handle a limited number of cells or preselected genes or part of data modalities on individual slices [20, 21], which also largely reduces their scope of applications in SRT studies as a rapid expansion in the amount and type of SRT data. Remarkably, none of the existing GRN analysis frameworks for SRT data, to the best of our knowledge, can effectively accommodate diverse characteristics of SRT data to infer spatial context-aware regulatory networks, advancing the understanding of cellular behavior in complex tissue microenvironments.

To address these issues, we propose Spatially Aligned Graph Transfer Learning (SpaGTL), a scalable generalized framework equipped with a novel large-scale cross-dimensional model, for the inference of context-specific spatial GRNs from SRT data with varying resolutions or modalities. In the core model, SpaGTL employs tailored structures for gene and cell/spot dimensions: gene-level graph transformers for modeling gene regulatory characteristics and cell/spot-level manifold-dominated variational autoencoder (VAE) for spatial graph representations, which are effectively concatenated by deep spatial distribution alignment. This allows optimally transferring two types of relational information—gene regulatory and cell/spot proximity—while flexibly incorporating spatial context and transcriptional variations in a self-supervised mechanism. From an application perspective, SpaGTL enables the inference of microenvironment variation-related regulatory modules (regulons) and the identification of derived functional regions and cell types. Particularly, to facilitate GRN inference with limited SRT data, SpaGTL is pretrained on a large-scale dataset encompassing approximately 100 million cells/spots to provide a GRN foundation for the fine-tuning predictions in a specific context. Moreover, SpaGTL refines the network regulators via cis-regulatory motif analysis for further improving the delineation of spatially regulatory heterogeneity.

To verify the ability of SpaGTL, we performed evaluation experiments to show the advantage of our model architecture and used a diverse panel of context-specific examples to reveal its broader application in real scenarios. When applied to the 10X Visium mouse brain dataset, SpaGTL distinguished domain-level spatial regulatory patterns, especially for spatially adjacent yet of the same anatomical structures. Applied to Slide-seqV2 mouse cerebellum dataset, SpaGTL exhibited context-aware to elucidate cellular-level regulatory architecture on the basis of spatial distributions of cell types and also imputed regulation signals in spots with absent expressions. Furthermore, SpaGTL has been extended to process multi-slice multi-stage *Drosophila* embryo Stereo-seq datasets, mapping the tissue function-specific 3D regulatory landscape and exploring critical regulator dynamics along cell differentiation from a 3D spatiotemporal perspective.

## Methods

### Overview of SpaGTL

**Figure 1:**
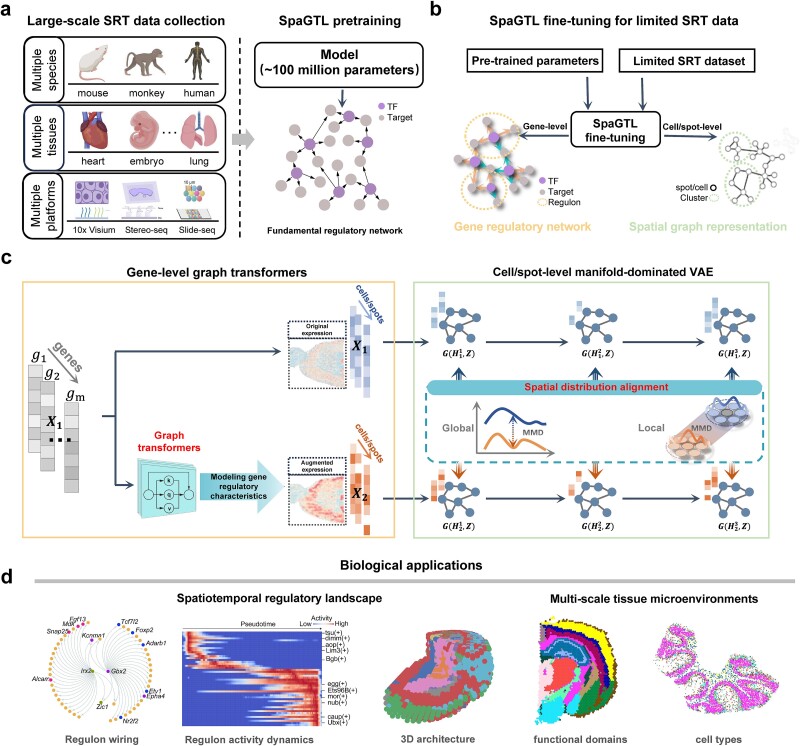
The schematic overview of SpaGTL. (a) a large-scale SRT data collection with 96 million spots/cells, spanning from different species, tissues and platforms, was used for pretraining the model and generating a fundamental GRN. (b) the fine-tuning on specific SRT datasets, with copying pretrained attention weights as the initial values, can be used for inference of context-specific regulatory networks resolving heterogeneous spatial microenvironments at different scales. (c) Outline of the core architecture. Original gene expression matrix serves as the initial training data (i.e. *X_1_*). Augmented expression matrix (i.e. *X_2_*) is generated through a graph-transformer block, which reconstructs expression values from potential regulated genes based on attention weight. In encoder-decoder, the alignment strategy constrains the learnable manifold structure (i.e. *Z*) in representation learning, which aligns global distributions from original and augmented views as well as their spatially local distributions over all hidden layers. (d) Biological applications for specific fine-tuning datasets, including regulon (i.e. regulatory module) inference, spatiotemporal regulatory heterogeneity analysis, functional domain detection, and cell type identification. 3D, three-dimensional; MMD, maximum mean discrepancy; TF, transcriptional factor.

We developed SpaGTL, a novel SRT analytical framework equipped with a large-scale cross-dimensional learning model (approximately 100 million parameters) that enables inference of context-specific spatial regulatory architecture. This is pretrained on an extensive collection of SRT data comprising ~100 million cells/spots, spanning diverse tissues and SRT platforms (Fig. 1a). This pretraining allows the model to gain a generalized foundation of spatial regulatory network, accommodating the complex patterns of gene relationships across diverse cell states. Subsequently, the fine-tuned SpaGTL facilitates the GRN inference for limited SRT datasets and also derives cell types or functional domains in scenarios characterized by heterogeneous regulatory programs (Fig. 1b).

The core of SpaGTL is a cross-dimensional deep learning model for effective learning and integration (Fig. 1c). It includes tailored neural network architectures for gene and cell dimensions, complemented by a deep alignment strategy. For gene dimension, taking the original expression data (i.e. ${X}_1\in{R}^{M\times N}$ with $M$ genes $\times N$ spots/cells) as input, SpaGTL utilizes attention-based graph transformers (gene graph denoted as $S\in{R}^{M\times M}$) to model gene regulatory characteristics and accordingly to generate augmented expression matrix (i.e. ${X}_2\in{R}^{M\times N}$). This is based on a generalized mathematical assumption that expression values of each gene can be reconstructed by the co-expressed genes [12]. For cell/spot dimension, utilizing initial data from gene dimension (i.e. ${X}_1,{X}_2\in{R}^{M\times N}$), SpaGTL introduces a novel manifold-dominated VAE to contrastively learn the spatial graph representation (cell graph denoted as $Z\in{R}^{N\times N}$) on a spatially aware manifold space. This spatial graph representation reflects both global and spatially local similarities between cells/spots derived from original and augmented views. Furthermore, SpaGTL employs a deep spatial distribution alignment strategy that captures and preserves data structures throughout embedding layers to integrate the two architectures into a cohesive cross-dimensional deep learning framework, which enables the simultaneous characterization of gene-level regulatory networks and cell/spot-level representations. The overall design is accomplished on completely unlabeled SRT data, allowing the inclusion of datasets without being limited to data with accompanying annotations, addressing a significant challenge in this field. Notably, the basic architecture remains consistent between pretraining and fine-tuning phases, while the objectives differ slightly due to the specific goals of each phase (see detailed Methods). During pretraining, the focus is on establishing a fundamental gene network. Thus, we enhance its biological relevance and interpretability by constraining it to the prior knowledge molecular network.

During fine-tuning phase, SpaGTL optimizes context-specific outputs—namely, attention weight $S$ for gene relationships and graph weight $Z$ for cell relationships—upon reaching model convergence; the optimal ones are used for downstream analytical tasks in two dimensions (Fig. 1d). The graph weight $Z$ facilitates the resolving of cell clusters in tissue, termed spatial domains or cell types for SRT data with different resolutions. The attention weight $S$, which indicates potential regulatory interactions among genes, is refined by excluding indirect target genes for each TF through regulatory sequence analysis using cisTarget [12]. This pruning enables the precise inference of GRNs, which are further categorized into regulons, each comprising a TF and its direct targets. Combining the regulatory modules and cell clusters, SpaGTL identifies critical network regulators that govern tissue microenvironmental heterogeneity specific to each tissue architecture. Note that the cross-dimensional fusion design facilitates SpaGTL transferring information over different dimensional graphs in a self-supervised manner, thus enhancing the identification of GRNs and cell types/spatial domains with improved biological correspondence and spatial coherence.

### Data collection and preprocessing

*Large-scale data* *for pretraining*: We collected human, monkey, and mouse SRT data from a variety of publicly available databases and resources, including STOmics, SOAR, SpatialDB, CROST, and the 10X Genomics website (available at 10X Genomics), as well as Census (available at Cellxgene). To ensure consistency across different datasets, we standardized the gene symbols and aligned them to a comprehensive gene list that included 31 053 entries. Our extensive dataset, which underwent rigorous quality control, consists of 96 700 729 cells/spots from 7367 tissue slices, spanning 365 samples across a diverse array of tissues, including the lung, skin, brain, liver, kidney, spinal cord, and embryo, and encompassing normal and various diseases such as pancreatic ductal adenocarcinoma, amyotrophic lateral sclerosis, non-small cell lung cancer, and hepatocellular carcinoma. The batch effect in pretraining can be neglected due to the parameter updating on a per-slice basis. This large-volume collection forms the basis for the pretraining of our SpaGTL model, providing a robust foundation for capturing intricate GRNs across a wide array of biological contexts and conditions. Additionally, we also collect network knowledge, composed of 3 729 929 protein–protein interactions and 3 592 299 GRNs from NicheNet [23] and STRING [24]. The prior gene network serves as the gold standard reference (denoted as matrix $A$ in the model) for location coding in the pretraining process. The normalized confidence score of gene network is used as the weight value of matrix $A$. Note that, gene symbols standardization may result in the loss of biologically significant information. For instance, certain genes might not map to standardized gene symbols due to platform-specific legacy identifiers or outdated annotations. Furthermore, species-specific or paralogous genes that do not align well with reference gene lists may be inadvertently excluded despite their functional relevance. To mitigate these issues, we employed comprehensive reference databases, such as ENSEMBL [25] and HGNC [26], to enhance gene coverage and mapping accuracy. Moreover, instead of using the intersection of genes across species or platforms, we opted for the union (i.e., 31 053 entries) to ensure that records of unmapped genes are preserved for future reassessment. For genes not included in the pretraining phase, their parameters can be initialized randomly, and adjustments can be made during the fine-tuning stage based on the corresponding SRT data.

*Specific SRT datasets for fine-tuning*: For specific studies, we selected datasets with varying technical and biological characteristics: 10X Visium mouse brain coronal data, Slide-seqV2 mouse cerebellar data, and Stereo-seq *Drosophila* embryo/larval data. These datasets were independently processed and intentionally excluded from the pretraining dataset. Each dataset involves the preprocessing of gene selection. For the 10X Visium data, we utilized the sc.pp.highly_variable_genes() function from the SCANPY package [27] to extract the top 3000 highly variable genes. For the Slide-seqV2 and Stereo-seq datasets, we relied on the marker gene lists provided in the original publications, which comprised 3272 and 2224 genes, respectively, for subsequent GRN inference. Then, we performed log transformation on the expression profiles after adding a pseudocount of 1 implemented in scanpy.pp.log1p(), which subsequently served as the input for SpaGTL. Note that the serial slices of *Drosophila* embryo data have been removed from batch effects from the original work [28]. Although the pretraining phase is limited to data from mouse, monkey, and human, the combination of pretraining and a fine-tuned architecture enables the model to effectively handle data from other species. First, we standardize the genes of the target species, extract the relationship parameters for genes in the pretrained gene list, and randomize the relationship parameters for genes that are not present in the pretrained list. The model is then fine-tuned using the given spatial transcriptomics (SRT) data, allowing recalibration of interactions between species-specific genes and the inference of GRNs.

### Spatially local graph construction from single slice or multi-slices dataset

In addition to gene measurements, SRT datasets also include the spatial locations of spots/cells or may include other modality information (i.e. histological image). Based on these, we construct a spatially local graph for a spatial-aware graph representation learning in the core model of SpaGTL.

First, we calculated the Euclidean distances between spots following Hu *et al*.’s preprocessing method [22] based on the available modalities in each data. Based on the Euclidean distance and the locational coordinates, we selected *k*-nearest neighbors for each spot (default *k* = 10 for 10X Visium datasets, 30 for Silde-seqV2, and Stereo-seq datasets). Generally, spot coordinates are 2D, i.e. x-y axes, for a single slice, while the coordinates may extend to 3D, i.e. x-y-z axes when serial slices are integrated. For 3D datasets, the selection of spatial neighbors is slightly adjusted to make SpaGTL better fit this type of data. We will project all the sections along the z-axis into a common 2D (i.e. x-y) plane and select those nearest neighbors in the adjacent slices as the fair neighborhood.

Then, we constructed the weighted adjacency graph ($B\in{R}^{N\times N}$, $N\ \mathrm{is}\ \mathrm{the}\ \mathrm{number}\ \mathrm{of}\ \mathrm{spots}/\mathrm{cells})$ which can reflect the spatially local structure of SRT data. Each weight was calculated based on principal component analysis (PCA) embeddings ($U\in{R}^{K\times N}$, $K\ \mathrm{is}\ \mathrm{the}\ \mathrm{number}\ \mathrm{of}\ \mathrm{top}$ PCs) of expression data as follows:


(1) \begin{equation*} {B}_{ij}=\frac{D_{ij}}{\sum_{i=0}^N{D}_{ij}}, where\ {D}_{i,j}=\mathit{\exp}\left(2-\frac{\left\langle{U}_i,{U}_j\right\rangle }{\left\Vert{U}_i\right\Vert \cdot \left\Vert{U}_j\right\Vert}\right) \end{equation*}

where ${B}_{ij}$ indicates the scaled similarity between spot *i* and *j* and $\left\langle{U}_i,{U}_j\right\rangle$ denotes the Euclidean distance in PCA latent space. $\left\Vert{U}_i\right\Vert$ and $\left\Vert{U}_j\right\Vert$ are the modules of vector ${U}_i$ and ${U}_j$, respectively.

### Building spatially aligned graph transfer learning

As a novel cross-dimensional transfer learning framework, SpaGTL encompasses three key components: graph transformers, a manifold-dominated VAE, and deep spatial distribution alignment. These components are integral to modeling gene regulatory characteristics, learning spatial graph representations, and ensuring integration of dimension-specific neural network architectures.

**Graph transformers for modeling gene regulatory characteristics**. Suppose we denote the original SRT expression as: ${X}_1\in{R}^{M\times N}$ (where $M$ is the number of genes and $N$ is the number of spots/cells). Taking it as input, SpaGTL utilizes graph transformers for modeling a learnable gene–gene relationship graph $S\in{R}^{M\times M}$ that can serve to generate augmented expression data ${X}_2\in{R}^{M\times N}$ for linking the cell/spot-level VAE architecture. This gene-level graph transformer is built as follows.

*Multi-head self-attention module:* Self-attention allows information to transfer across different genes by which the original data can be reconstructed as augmented data. Here, we use a multi-head attention mechanism, and in $\xi $’th self-attention layer, we obtain the attention weight ${S}^{\xi } $ and the augmented data ${X}_2^{\xi +1} $ as:


(2) \begin{equation*} {\displaystyle \begin{array}{c}{X}_2^{\xi +1}= concact\left({S}_1^{\xi }{V}_1^{\xi },\dots, {S}_t^{\xi }{V}_t^{\xi },\dots, {S}_{\Gamma}^{\xi }{V}_{\Gamma}^{\xi}\right)\end{array}} \end{equation*}

(3) \begin{equation*} {\displaystyle \begin{array}{c}{S}_t^{\xi }= Normalization\left( softmax\left(\frac{Q_t^{\xi}\cdotp{\left({K}_t^{\xi}\right)}^T}{\sqrt{d_t}}\right)+\mu A\right),\\t=1,\dots, \Gamma; \xi =0,\dots, \mathrm{Y}\end{array}} \end{equation*}

where ${Q}_t^{\xi }={X}_2^{\xi }{W}_{t,Q}^{\xi}\in{R}^{M\times{d}_t},{K}_t^{\xi }={X}_2^{\xi }{W}_{t,K}^{\xi}\in{R}^{M\times{d}_t},{V}_t^{\xi }={X}_2^{\xi }{W}_{t,V}^{\xi}\in{R}^{M\times{d}_t}$ respectively represent the query, key, and value of the $t$’th head in $\xi$’th layer; while ${d}_t$ denotes the dimensionality of the queries and keys. $concact\left(\ast \right)$ denotes concatenation along the rows. For simplicity, we denote the original expression ${X}_1$ as ${X}_2^0$ and augmented expression as: ${X}_2^{\mathrm{Y}}$($\mathrm{Y}\ge 1)$ in formulas (2–3). ${S}_t^{\xi }$ is the attention weight of the $t$’th head in $\xi$’th layer. The average attention weight $S={\sum}_{\xi =0}^{\mathrm{Y}}{\sum}_{t=1}^{\Gamma}{S}_t^{\xi }/\left(\Gamma +\mathrm{Y}\right)$ is used to measure overall gene–gene relationships, $\Gamma$ is the number of heads. Formula (3) utilizes the query (${Q}_t^{\xi }$) and key (${K}_t^{\xi }$) matrices of the multi-head self-attention mechanism to compute the attention weight (${S}_t^{\xi }$) without involving the value matrix (${V}_t^{\xi }$). The value matrix ${V}_t^{\xi }$ serves a distinct purpose in the multi-head self-attention mechanism: it fuses the attention weights ${S}_t^{\xi }$ to generate the output matrix ${X}_2^{\xi +1}$ for the $\xi$-th self-attention layer, as specified in formula (2). During the pretraining phase, the loss function is designed to construct a generalized fundamental GRN by constraining the weight to prior gene network knowledge (i.e. matrix $A$). To achieve this, the parameter $\mu$ should only be set to non-zero in pretraining phase and default to 0.2, which controls biological implication of inferred networks $S$ based on the prior gene network knowledge (Supplementary Fig. S1). In contrast, during the fine-tuning phase, $\mu$ is set to zero as the focus shifts to optimizing context-specific outputs, such as gene–gene attention weights and cell/spot graph weights. At this stage, the loss function prioritizes accuracy in these specific tasks, aligning with the context-dependent goals of fine-tuning.

*SVD-based positional encodings*: We constructed an undirected gene graph using the collection of prior-knowledge gene interactions (see Data collection). We decomposed its adjacency matrix (i.e. $A\in{R}^{M\times M}$) through Singular Value Decomposition (SVD) to obtain the positional encodings.


(4) \begin{equation*} {\displaystyle \begin{array}{c}A=U\Sigma{V}^T=\left(U\sqrt{\Sigma}\right){\left(V\sqrt{\Sigma}\right)}^T= \tilde{U}{\tilde{V}}^T,P=\left(\tilde{U}\Big\Vert \tilde{V}\right){W}_{PE}\end{array}} \end{equation*}

where $U,V\in{R}^{M\times r}$ matrices contain the largest $r$ left and right singular vectors as columns, respectively, corresponding to the top $r$singular values in the diagonal matrix $\Sigma \in{R}^{r\times r}$. $\Big\Vert$ denotes concatenation along the columns. ${W}_{PE}\in{R}^{2r\times N}$ is a learned projection matrix and $P\in{R}^{M\times N}$ matrix can be used as positional encodings.

**Manifold-dominated VAE for learning spatial graph representation**. Given the original and augmented data as input, SpaGTL builds a VAE on manifold space to learn spatial-aware graph representation for cell/spot dimension. This architecture consists of an encoding component, a decoding component, and deep self-expressive component.

*Encoding component*: We employ the encoder network to extract representations exclusively from the data of original and augmented expressions. The specific representations denoted as ${H}_1^{(i)}$ and ${H}_2^{(i)}$ of gene expression in the $l$-th layer encoder as follows:


(5) \begin{equation*} {\displaystyle \begin{array}{c}{H}_1^{(i)}={\phi}_i\left({W}^{(i)}{H}_1^{\left(i-1\right)}+{b}^{(i)}\right),i=1,\dots, l\end{array}} \end{equation*}

(6) \begin{equation*} {\displaystyle \begin{array}{c}{H}_2^{(i)}={\phi}_i\left({W}^{(i)}{H}_2^{\left(i-1\right)}+{b}^{(i)}\right),i=1,\dots, l\end{array}} \end{equation*}

where ${\phi}_i$ represents the activation function in the $i$-th layer. ${W}^{(i)}$ denotes the weight matrix. ${b}^{(i)}$ corresponds to the bias term. The original and augmented data matrices (i.e. ${X}_1$ and ${X}_2$) are respectively simplified as ${H}_1^{(0)}$ and ${H}_2^{(0)}$ in formulas (5–6).$l$ is the number of encoding layers.

*Decoding component*: Following the encoding process, the decoder network is used for data reconstruction in which the $i$-th layer’s representations are denoted as follows:


(7) \begin{equation*} {\displaystyle \begin{array}{c}{H}_1^{(i)}={\psi}_i\left({W}^{(i)}{H}_1^{\left(i-1\right)}+{b}^{(i)}\right),i=l+1,\dots, L\end{array}} \end{equation*}

(8) \begin{equation*} {\displaystyle \begin{array}{c}{H}_2^{(i)}={\psi}_i\left({W}^{(i)}{H}_2^{\left(i-1\right)}+{b}^{(i)}\right),i=l+1,\dots, L\end{array}} \end{equation*}

where ${\psi}_i$ represents the activation function of the $i$-th decoder layer. $L$ is the total number of encoder-decoder layers, equal to $l\times 2$. ${\phi}_i,{\psi}_i,{W}^{(i)}\ \mathrm{and}\ {b}^{(i)}$ are shared within each layer from original and augmented views.

*Deep self-expressive component*: This module is the key to performing VAE on manifold space, which can better reflect complex SRT data structure. In this step, we use a view-unified manifold structure (i.e. $Z\in{R}^{N\times N}$) to constrain the encoding and decoding operations. The manifold structure not only reduces the influence of data sparsity on representation learning but also enables the model to learn consistent graph representations from the original and augmented views. The loss function for this goal is defined as:


(9) \begin{equation*} \displaystyle \begin{array}{c}{\mathcal{L}}_{manifold}=\frac{1}{L}\sum\limits_{i=1}^L{\left\Vert{H}_1^{(i)}\left(I+\alpha B\right)-{H}_1^{(i)}Z\right\Vert}_F^2/\left\Vert{H}_1^{(i)}\right\Vert\\\qquad\ \ +{\left\Vert{H}_2^{(i)}\left(I+\alpha B\right)-{H}_2^{(i)}Z\right\Vert}_F^2/\left\Vert{H}_2^{(i)}\right\Vert \end{array} \end{equation*}

where $B$ is the spatially local graph established in the previous description. Parameter $\alpha$ is tunable and can be manually set according to the obtained outcomes, which controls the impact of spatial local structure on spatial graph representation learning and GRN inference (Supplementary Fig. S2).

**Deep spatial distribution alignment**. To effectively link the neural network architectures for gene and cell dimensions, we design a strategy for the alignment of representational distributions respective from original and augmented views. This is termed “deep spatial distribution alignment”, which can characterize and preserve graph structures throughout each embedding layer during training.

*Aligning the global distribution of view-specific representations*: As the distribution of spots should reflect clustering assignments, we contrast and expect to align distributions of the view-specific representations. This can facilitate the preservation of manifold structure $Z$ for achieving consistent clustering in a global perspective. We define the loss function for quantifying global distribution differences using Maximum Mean Discrepancy (MMD) [29].


(10) \begin{equation*} {\displaystyle \begin{array}{c}{\mathcal{L}}_{alig{n}_{GD}}=\frac{1}{L}\sum\limits_{i=1}^L MMD\left({H}_2^{(i)},{H}_1^{(i)}\right)\end{array}} \end{equation*}

The MMD metric is defined as:


$$ MMD\left(X,Y\right)={\left\Vert \frac{1}{N}\sum_{i=1}^N\Psi \left({x}_i\right)-\frac{1}{N}\sum_{j=1}^N\Psi \left({y}_j\right)\right\Vert}_H^2 $$

(11) \begin{equation*} {\displaystyle \begin{array}{c}=\frac{1}{N^2}\sum\limits_{i=1}^N\sum\limits_{j=1}^NK\left({x}_i,{x}_j\right)-\frac{2}{N^2}\sum\limits_{i=1}^N\sum\limits_{j=1}^NK\left({x}_i,{y}_j\right)+\frac{1}{N^2}\sum\limits_{i=1}^N\sum\limits_{j=1}^NK\left({y}_i,{y}_j\right)\end{array}} \end{equation*}

where $\Psi \left(\cdot \right)$ function is used to project the data into a regenerative Hilbert space. ${\left\Vert \cdot \right\Vert}_H^2$ measures the distance of data in this regenerated Hilbert space. $K\left(x,y\right)={e}^{-\frac{{\left\Vert x-y\right\Vert}^2}{2}}$ denotes the Gaussian kernel function to determine the distance between vectors.

*Aligning the spatially local distribution of view-specific representations*: Considering the influence of spatial context, spatial neighbors of each spot tend to belong to the same clustering assignments or share similar transcriptional patterns, whether from the original view or augmented view. We write the loss function for local structural alignment as follows:


(12) \begin{equation*} {\displaystyle \begin{array}{c}{\mathcal{L}}_{alig{n}_{SLD}}=\frac{1}{\left(L\ast N\right)}\sum\limits_{i=1}^L\sum\limits_{k=1}^N MMD\left({\left({H}_2^{(i)}\right)}_k^{Nei},{\left({H}_1^{(i)}\right)}_k^{Nei}\right)\end{array}} \end{equation*}

where ${\left({H}_1^{(i)}\right)}_k^{Nei},{\left({H}_2^{(i)}\right)}_k^{Nei}$indicate the $i$-th layer’s representations of the spatial neighbor spots for spot *k*, respectively from the original and augmented data.

*Contrasting view-specific representations*: The distribution alignment of representations does not prevent data drift of each spot. Here, we contrast view-specific representations to solve this problem. We write the contrast loss function as:


(13) \begin{equation*} {\displaystyle \begin{array}{c}{\mathcal{L}}_{CL}=\frac{1}{L}\sum\limits_{i=1}^L{\left\Vert{H}_1^{(i)}-{H}_2^{(i)}\right\Vert}_F^2\Big/\left\Vert{H}_1^{(i)}+{H}_2^{(i)}\right\Vert \end{array}} \end{equation*}

### Optimizing deep neural network training

Combined with the losses of VAE for original and augmented expression data (i.e. ${\mathcal{L}}_{ELBO}^1,{\mathcal{L}}_{ELBO}^2$), the overall loss function of SpaGTL can be denoted as formula (14).


(14) \begin{equation*} {\displaystyle \begin{array}{c}{\mathcal{L}}_{overall}=\underset{manifold- dominated\ VAE\ loss}{\underbrace{\left({\mathcal{L}}_{ELBO}^1+{\mathcal{L}}_{ELBO}^2+{\lambda}_1{\mathcal{L}}_{manifold}\right)}}+\underset{cross- dimension\ loss}{\underbrace{\lambda_2\left({\mathcal{L}}_{CL}+{\mathcal{L}}_{alig{n}_{GD}}+{\mathcal{L}}_{alig{n}_{SLD}}\right)}}\end{array}} \end{equation*}

where the tunable parameter ${\lambda}_1$ and ${\lambda}_2$ can be manually set, which controls the spot-level graph representation learning and are stable to the interference of the model performance within a certain range based on the sensitivity analysis of the parameters (Supplementary Fig. S3).

We performed ablation experiments in the pretraining phase and verified that each component could effectively improve the performance of the model (Supplementary Fig. S4). By minimizing this objective function, the gene–gene relationships are extracted over the learned attention weight within the given spatial transcriptome passed into the model and benefit from the foundational knowledge learned during pretraining; the learned graph representations can also be used for cell clustering that resolves the tissue architecture.

### Pretraining on a large-scale SRT data

*SpaGTL architecture*: The core model of SpaGTL is composed of gene-level graph transformers and cell/spot-level manifold-dominated VAE. For graph transformers, there are three transformer encoder units, each composed of a full dense multi-head self-attention layer and feed-forward neural network layer with the following parameters: input size of 31 053 and five attention heads. For manifold-dominated VAE, the number of encoder-decoder layers is set to two by default. The encoder and the decoder of two layers are sufficient to capture the complex data structure in spatial transcriptomics and, meanwhile, maintain the model’s simplicity. The number of neurons in the hidden layer is set to 128, and the number of neurons in the latent layer is set to 10, which exhibits superior performance throughout all experiments performed in manuscript.

*SpaGTL pretraining*: We set learning rate to 1e-3, learning scheduler to linear with warmup, optimizer to Adam with weight decay fix, and warmup steps to 10 000. To mitigate potential batch effects among different slices, we divided a single slice into multiple token datasets, enabling coverage of all spots/cells, with each token data set containing 3000 spots/cells. We trained the model on each individual token data until convergence. We subsequently used the obtained model parameters as initial settings for training on the next token data, thereby refining the learned gene relationship matrix $S$. The above training process is repeated until all the collected datasets have been trained. Then, the token data order was shuffled to train the model until the obtained gene relationship matrix $S$ became stable. Note that the broad diversity of the pretraining dataset significantly reduces the risk of model overfitting to any dataset. We also adopted a per-slice training and random permutation strategy, wherein model parameters are updated independently for each slice. This approach minimizes the influence of individual datasets on the overall model. To further prevent overfitting, regularization techniques such as weight decay were applied, and a linear learning rate scheduler with warmup was employed to ensure stable training dynamics. Therefore, the large-scale pretraining does not overfit certain specific datasets.

### Spatial clustering

SpaGTL employs the learnt cell relation matrix $Z$ to cluster cells using the Leiden [30] community detection algorithm, as implemented by scanpy.tl.leiden() in SCANPY. We annotated the identified cell clusters to enhance the resolution of spatial regulatory patterns. The “resolution” parameter in both Leiden algorithms was adjusted to match the number of annotated structures, if available, or was manually defined based on prior anatomical knowledge. For datasets lacking prior information, the resolution for the Leiden algorithm was manually selected.

For the DLPFC dataset, clustering analyses were performed under scenarios involving 1000, 2000, 3000, 4000, and 5000 highly variable genes. For each scenario, the Adjusted Rand Index (ARI) was computed to evaluate the clustering accuracy [31–34], and the runtime of each method was recorded to assess computational efficiency. These results were benchmarked against competing methods to provide a comprehensive evaluation of performance.

For the 10X Visium dataset, we manually annotated the clustering results as spatial domains, referencing the anatomical diagram from the Allen Brain Atlas [35, 36]. In the Slide-seqV2 dataset, we utilized cell labels from cerebellum scRNA-seq data to annotate the clusters as specific cell types. For the Stereo-seq dataset, we directly applied the annotations from the data source to classify the clustering results into spatial domains and cell types.

### Regulon identification and activity calculation

In the co-expression gene network $S$, gene interactions are solely identified on correlations of gene expressions, which may include direct and indirect relationships. To filter out those indirect or low-confidence interactions, we thus trimmed the network using the motif analytical tool cisTarget implemented in pySCENIC package [37]. Given the gene set, cisTarget collects and annotates the TF-binding motifs that are significantly overrepresented surrounding the transcription start site (TSS) of the genes. Those putative targets for each TF are retained which, shows the enrichment of any motif of the corresponding TF. In this way, the network is refined as a collection of regulatory subnetworks, i.e. regulons, which connect each TF and the putative direct targets.

Subsequently, we calculated the activity of each regulon (including a TF and its target genes) on each spot using AUCell. AUCell calculates the enrichment of the genes in regulon as an area under the recovery curve across the ranking of all genes in a particular spot, where genes are ranked by their expression values [12]. This approach is effective to measure whether a critical subset of the target genes show high expressions, that is, as a result of an active regulon governing the cell. Using AUCell, we quantified the regulon activity to further investigate the spatial regulatory patterns spanning resolved tissues.

### Domain/cell type-specific regulons identification

Domain/cell type-specific regulons are identified using the FindAllMarkers() function from the Seurat R package, leveraging the AUCell matrix. These identified regulons are subsequently filtered to retain only those with high specificity. For the 10x Visium datasets, domain/cell type-specific regulons are filtered based on an Area Under the Receiver Operating Characteristics (AUROC) greater than 0.75 and an average log_2_ fold change exceeding 0.25. For Slide-seqV2 and Stereo-seq datasets, the filtering criterion for domain/cell type-specific regulons is set at a log_2_ fold change greater than 0.25.

### Performance evaluation in inference of GRNs

The general evaluation for reverse-engineering regulatory networks from expression profiles is performed to measure the similarity between the predicted edges and the “ground truth” ones. For this purpose, some metrics are often adopted, e.g. AUROC and Jaccard index, based on the ranking of all the edges due to the predicted weights. In the work, we would like to evaluate how well the GRNs inference methods work on SRT datasets which include spatial information besides expression measurements; this encourages us to take additional account of whether the inferred networks show spatial dependency underlying tissue organization. For example, tissue consists of various cell types that are spatially arranged with regularity, specifying biological functions as different compartments. While the locations within the same microenvironments may be more transcriptionally similar than those from areas distinct and far apart. Such transcriptional differences suggest various regulatory programs characterizing tissue architecture. In this consideration, we divide the overall network detected from each method as a collection of regulons (TFs and their targets) which may serve as the basic regulation units. With the defined subnetworks, we measure at each location whether a particular regulation is activated according to the expression levels of the target genes (see Regulon identification and activity calculation). In this way, we take other criteria to evaluate if the identified regulons exhibit spatial activity patterns. The detailed evaluation pipeline is described in the following.

**Evaluation of the overall network**. We evaluate the GRN prediction performance on simulated and *Drosophila* Stereo-seq datasets using similar criteria as in Pratapa *et al*.’s work [38], including prediction accuracy, running stability, and time efficiency.

*Prediction accuracy*: We utilized AUROC and AUPRC to measure the prediction accuracy. For simulated datasets, we executed each algorithm 50 times and calculated AUROC and AUPRC values for each run. For real *Drosophila* datasets, in order to calculate the value of AUROC, we used the transcription factor-gene interaction data in The *Drosophila* Interactions Database [39] (DroID, http://www.droidb.org/) as the real label. The data contains a total of 39 451 pairs of different gene regulatory relationships. We only used 39 355 pairs of regulatory relationships detected by ChIP-seq as the real background label for calculating AUROC.

*Running stability*: In the repeated simulated experiments, we evaluated if the inferred networks from each stochastic method changed from one run to another. In every run, the edges are ranked by predicted weights. Then, comparing the previous and the current results, we computed the Spearman’s correlation over the lists of all the ranked edges and also used the Jaccard index to measure the overlap in the top-*k* edges. *k* represents the number of edges in the true network.

*Runtime efficiency*. For this purpose, we varied the numbers of genes or samples to randomly generate a series of sub-datasets from *Drosophila* E14 dataset. To make fair comparisons, when we performed the experiments with the varied number of genes, we fixed the number of samples (e.g., with 2000 samples) and vice versa (e.g., with 1000 genes). Methods were tested on a machine with one eight-core Intel i7-10700HQ CPU addressing 64 GB RAM and one NVIDIA GeForce RTX 3060 GPU addressing 12 GB RAM.

**Evaluation of the regulon activity**: We evaluated the spatial patterns of regulons on real datasets in aspects of spatial autocorrelation and specificity.

*Spatial continuity*: Based on the spatial coordinates, we used the Rfast2::moranI() function implemented in R to compute Moran’s I coefficient for each regulon.

*Regulon specificity*: Depending on the data annotation, we used Seurat::FindAllMarkers() to filter the differentially active regulons and obtained their log_2_FC values for each domain/cell type. We then used the (average) log_2_FC to evaluate the specificity of regulatory patterns of the regulons detected by each method.

### Pseudo-trajectory and functional enrichment analysis, network visualization

We extracted the testis subsection on the third larvae stage and performed RNA velocity analysis (using Python package dynamo [40]) to obtain the spatiotemporal ordering (i.e. pseudotime). We performed functional enrichment analysis on the gene members of regulons using the clusterProfiler R package [41].

We used Cytoscape [42] to visualize the inferred GRN and to highlight the genes with a set of enriched functional terms of interest.

## Results

### Benchmarking of fine-tuning SpaGTL on simulated SRT data

**Figure 2:**
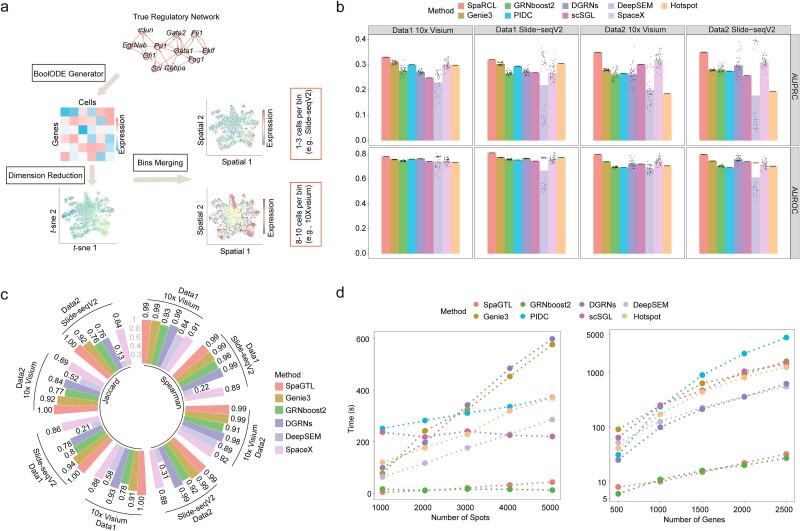
Benchmarking of fine-tuning SpaGTL against existing GRN inference methods. (a) Simulation data generation process. The process initiates with the generation of expression data at single-cell resolution using the BoolODE model. Then, this data is projected into *t*-distributed stochastic neighbor embedding (*t*-SNE) space [69], where several proximal cells in the embeddings are binned into a spot. (b) Evaluations of regulatory network inference accuracy. This panel shows the area under the receiver operating characteristic (AUROC) and area under the precision-recall curve (AUPRC), calculated between the inferred results and the ground-truth networks. Each method was applied 50 times to each dataset. (c) Method stability comparison. This panel compares the consistency of predicted outcomes from each method across the 50 repeated trials. Metrics used include Spearman coefficient and Jaccard coefficient. PIDC, scSGL, and hotspot, three methods that do not involve stochastic processes are excluded from this comparison. (d) Computational efficiency comparison. The comparison assesses the impact of both gene and spot quantities on the computational efficiency of the methods.

We assessed the efficacy of fine-tuning SpaGTL in predicting GRNs using four simulated SRT datasets. The data is first generated based on real networks at the single-cell level. The spot-level values and location information are then simulated by aggregating the embeddings at various resolutions (Fig. 2a, Supplementary Figs S5 and S6, and Supplementary Note 1.1 for details). The simulation makes these data closely mimic the SRT data characteristics, enabling fine-tuning with the pretrained weights. With these datasets, we benchmarked SpaGTL against two SRT-specific inference methods (i.e. SpaceX [20] and Hotspot [21]) and six established scGRN inference methods (i.e. Genie3 [43] and GRNboost2 [44], PIDC [13], scSGL [45], DeepSEM [14] and DGRNs [46]). To minimize testing variability, each method was executed 50 times per dataset. The prediction accuracy of each trial was quantified using Area Under the Receiver Operating Characteristic (AUROC) and Area Under the Precision-Recall Curve (AUPRC), while stability was assessed using the Spearman and Jaccard coefficients (details in Methods). For each metric, a value closer to 1 indicates good performance in this term.

Our analysis revealed that SpaGTL consistently achieved high prediction accuracy and significantly outperformed the competing methods across all datasets evaluated (Wilcoxon signed-rank test *P* < .0001; Fig. 2b). Besides, SpaGTL’s fine-tuning exhibited great stability in multiple tests, compared to other deep-learning-based methods (i.e., DeepSEM and DGRNs) and those dependent on sampling (i.e., SpaceX, Genie3, and GRNboost2) (Fig. 2c). Furthermore, we compared the computational efficiency of all methods, considering variations in the number of samples and genes in the datasets. We can see that SpaGTL shows stable and low time consumption when the number of spots varies, and the growth trend remains relatively slow when the number of genes increases, highlighting SpaGTL’s computational efficiency in fine-tuning compared to other *de novo* inference methods (Fig. 2d). Furthermore, we conducted a comparative analysis of spatial domain identification methods using the dorsolateral prefrontal cortex (DLPFC) datasets (Supplementary Fig. S7). The results indicate that SpaGTL is more efficient and accurate in identifying spatial structures compared to other competing methods.

Considering the metrics of inference accuracy, stability, and computational efficiency, SpaGTL consistently exhibits superior performance under diverse conditions. Therefore, employing SpaGTL to infer spatial regulatory networks and spatial clustering represents a more advantageous approach compared to existing mainstream methods.

### SpaGTL refines mouse brain anatomical structures from regulatory differences

**Figure 3:**
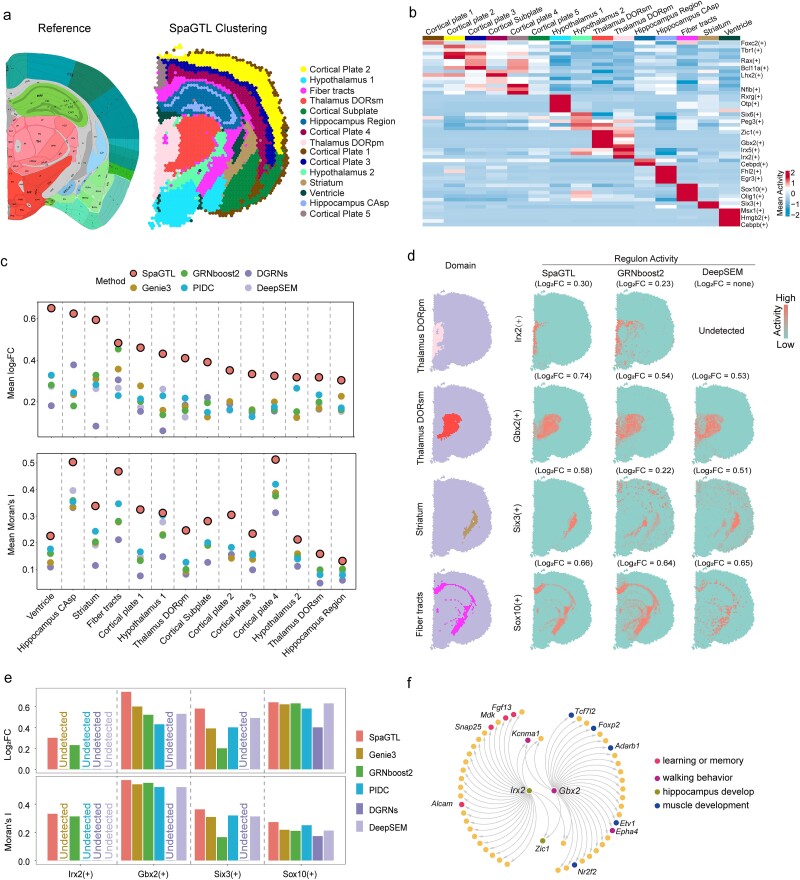
Exploration of domain-level regulatory patterns on 10X Visium mouse brain coronal data. (a) the clustering identified from fine-tuning SpaGTL is annotated based on the Allen brain reference atlas anatomical diagram. (b) Regulon activity heatmap. Each column presents a spatial domain as annotated in (a) and each row corresponds to a regulon which is denoted by its transcriptional factor, e.g. transcriptional factor(+). (c) Regulon pattern comparison analysis. For each marker regulon, log_2_FC (log2 fold change) is used to assess its activity specificity against all the other regions, and Moran’s I statistic is employed to measure its spatial continuity within the focused domain. (d) Illustration of representative marker regulons. The corresponding domains are shown on the left, and on the right, display the *in situ* staining activities of the marker regulons from SpaGTL, GRNboost2, and DeepSEM, presented sequentially. (e) Barplots for quantifying the patterns of the selected marker regulons from (d). A bar is replaced as “undetected” if the method did not infer this regulon. (f) Network topologies of Irx2(+) and Gbx2(+) regulons.

We fine-tuned SpaGTL on a real SRT dataset, the 10X Visium mouse brain coronal data, to analyze the regulatory patterns underlying spatial functional areas using the highly variable gene set. The functional regions, often defined as spatial domains, were not previously given but identified through sample clustering using the representation and subsequently annotated according to anatomical reference from the Allen Brain Atlas [35, 36] (Fig. 3a). As verified against the reference, SpaGTL demonstrated higher consistency in detecting corresponding regions compared to other domain detection methods (Supplementary Fig. S8). This also suggests the presence of different regulatory patterns among spatial areas of various functions.

For this, we pruned the SpaGTL-tuned gene network into a variety of regulons and computed the regulon activity score for each regulon for each spot (detailed in Methods). It is computed based on expression of the included target genes, allowing us to identify regulons with high activities. This analysis revealed the domain-wise difference and spatial continuity in regulatory patterns, fine-tuned by SpaGTL among various regions (Fig. 3b). For example, in the cortex, SpaGTL identified Lhx2(+), Rax(+), and Bcl11a(+) regulons which exhibited gradual variations and changes from superficial to deep layers (e.g. from Cortical plate 1 to 5). To further quantify such patterns, we employed the Moran’s I statistic and the log-base 2 of fold change (log_2_FC) to measure respective spatial correlation and biological differences of all identified regulons (see Methods). The higher values indicate regulons with better biological interpretations, accounting for spatial and regional coherence [47, 48]. Additionally, we conducted comparative analyses to other methods, which demonstrated that SpaGTL significantly outperformed the competing methods in capturing spatial regulatory modules from SRT data (Wilcoxon signed-rank test, $P=1.88\times{10}^{-21}$ for Moran’s I and $P=6.67\times{10}^{-17}$ for log_2_FC; Fig. 3c).

We subsequently selected four representative marker regulons of four spatially adjacent regions (i.e. Thalamus DORsm, Thalamus DORpm, striatum, and fiber tracts) as examples. These regulons, ever validated for their significant functions in each region [49–52], show notable regional specificity in our results (Fig. 3d, e). Particularly, the Irx2(+) and Gbx2(+) regulons showed high activities in respective Thalamus DORpm and Thalamus DORsm, two spatially proximate yet functionally distinct thalamic regions, exhibiting better spatial patterns compared to those from other methods (Fig. 3d, e). Their biological relevance of these regulons was confirmed through enrichment analysis; Gbx2(+) was enriched in processes related to muscle activity, whereas Irx2(+) was associated with the hippocampus and memory (Fig. 3f), aligning with the functional distinctions between the thalamus subdomains [53]. Notably, only SpaGTL and GRNboost2 identified the Irx2(+) regulon, in which the TF presents relatively low expression, indicating that SpaGTL presents more sensitive to uncover regulons with subtle expression levels (Supplementary Fig. S9). This fine-tuning on a 10-Visium slice demonstrates that SpaGTL excels in low-resolution SRT datasets, uncovering domain-level regulatory differences, even between regions of the same anatomical structure.

### SpaGTL enables identifying spatially colocalized cell types and key regulatory modules in high-resolution mouse cerebellum dataset

**Figure 4:**
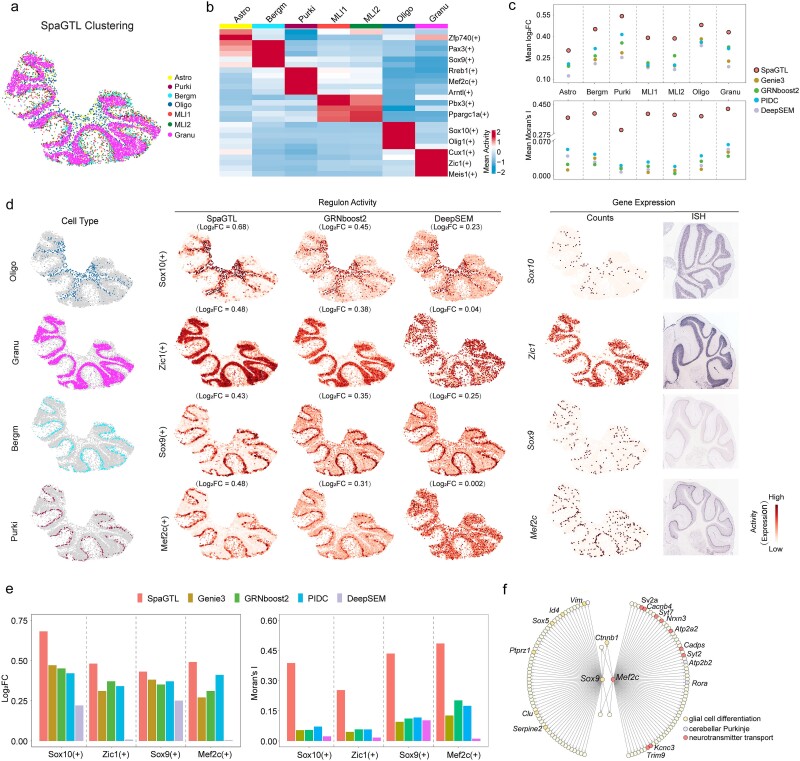
Investigating regulatory patterns among different cell types through slide-seqV2 mouse cerebellum dataset. (a) SpaGTL’s clustering on slide-seqV2 data is annotated based on single-cell marker genes [11]. (b) Regulon activity heatmap. (c) Regulon pattern comparison analysis on cell-type marker regulons from the inferred outcomes by each method. (d) Spatial patterns of the selected marker regulons or corresponding TFs. These ISH data are obtained from Allen’s brain atlas. (e) Barplots for quantifying the regulatory patterns of the selected marker regulons from (d). (f) the network topologies of Sox9(+) and Mef2c(+) regulons. Astro, astrocytes; Bergm, Bergmann; Granu, granule; MLI1, molecular layer interneurons 1; MLI2, molecular layer interneurons 2; oligo, oligodendrocytes; Purki, Purkinje.

We utilized Slide-seqV2 mouse cerebellar data, which offers near-cellular resolution expression profiles, to explore differential cell-type regulation patterns. Cell types were determined through sample clustering and annotated using marker genes derived from scRNA-seq data [11]. This analysis categorized the cells into four neuron types, i.e. Molecular Layer Interneurons 1 (MLI1), Molecular Layer Interneurons 2 (MLI2), granule cells, and Purkinje cells, and three non-neuron types, i.e. Bergmann glia, oligodendrocytes, and astrocytes (Supplementary Fig. S10). These cell types are organized mainly according with to cerebellum layered structure [54, 55], typically exhibiting well-defined and spatially continuous distributions, as depicted in Fig. 4a.

Given the spatially distributed architecture, we anticipated distinct regulatory patterns reflecting cellular differences and spatial correlations facilitated by SpaGTL’s fine-tuning. We accordingly mapped the regulon activities across these annotated cell types (Fig. 4b), which effectively outlines the heterogeneous regulatory programs previously validated in literature on the basis of cell-type context. For instance, the Sox10(+) regulon, highly active in oligodendrocytes, is linked to cell differentiation of oligodendrocytes [49], while Zic1(+), specific to granule cells, is associated with their development and maturation [56]. Furthermore, we quantified and compared the regulons identified by SpaGTL with those detected by competing methods (see Methods). In both Moran’s I and log_2_FC, regulons detected by SpaGTL stand out with notably higher values (Wilcoxon signed-rank test, $P=1.04\times{10}^{-11}$ for Moran’s I and $P=2.97\times{10}^{-17}$ for log_2_FC), indicating their regulatory patterns of enhanced spatial and biological coherence (Fig. 4c). These remarkable differences highlight SpaGTL’s advantage in delineating spatial regulatory modules for high-resolution SRT data.

To provide a more intuitive illustration, we selected the marker regulons specific to four cell types yet mainly localized in different layers, i.e., oligodendrocytes, granule cells, Bergmann glia, and Purkinje cells (Fig. 4d). The selected regulons can be detected by all methods involved; however, SpaGTL’s results demonstrated more precise in spatial regulatory activities, as stained faithfully corresponding to the cell distributions with higher specificity and continuity (Fig. 4e, Supplementary Fig. S11). In particular, the regulon activity facilitates the imputation of cellular regulatory signals, especially at those points where expression of relevant TFs is absent (Fig. 4d), since activity is calculated based on the expression of TF and the target genes, greatly alleviating data sparsity in high-resolution datasets. These also indicate the TF-target interactions predicted by SpaGTL are more context-specific, even for the spatially colocalized Purkinje and Bergmann glia. For Purkinje neurons, *Mef2c* serves as a marker TF and *Sox9* for Bergmann glia cells. SpaGTL inferred their target genes, many of which are enriched in cell type-specific biological processes such as the neurotransmitter transport pathway for Purkinje cells [57] and the glial cell differentiation pathway for Bergmann glia [58] (Fig. 4f). Despite the complex cell compositions in tissue spatial architecture, the context-aware SpaGTL ensures the delineation of cellular regulatory heterogeneity with reliable biological correspondence.

### Exploring critical regulatory modules along *Drosophila* 3D spatiotemporal variations

**Figure 5:**
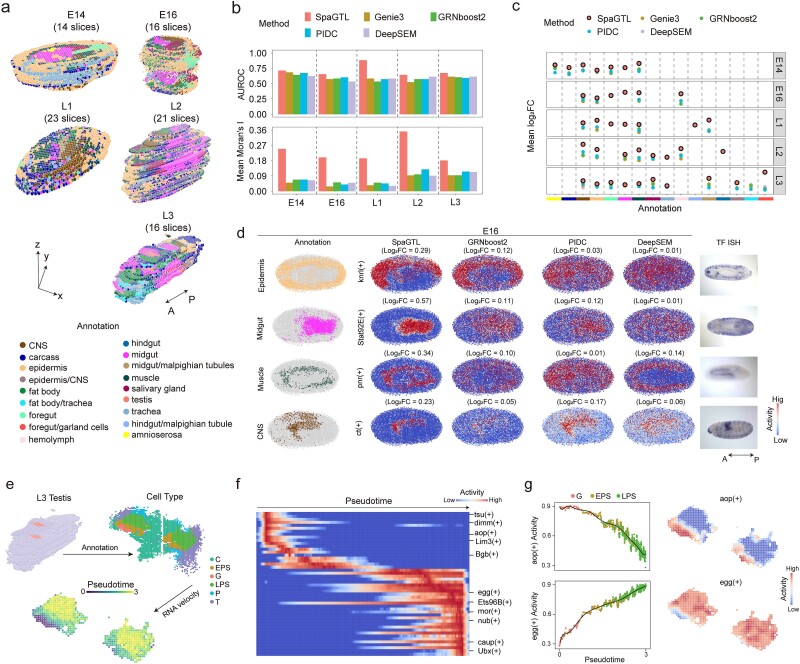
Exploration of spatiotemporal regulatory patterns on stereo-seq *drosophila* 3D data. (a) Data collection overview. Five multi-slice datasets encompass *drosophila* embryonic (i.e. E14 and E16) and larval (i.e. L1, L2, and L3) stages with the domain-level annotations provided originally [28]. (b, c) AUROC, Moran’s I, and Log_2_FC values are calculated to assess the prediction outcomes from different methods. (d) Spatial activity patterns of representative marker regulons detected by different inference methods for various embryonic structures. (e) Annotation and RNA velocity analysis of the testis from L3 transverse section. (f) Regulon activity patterns along the spatiotemporal axis. (g) Spatiotemporal patterns of egg(+) and aop(+).A–P, anterior–posterior; C, somatic cyst cells; EPS, early primary spermatocytes; G, spermatogonia; LPS, late primary spermatocytes; P, pigment cells; T, terminal epithelium precursor cells.

We then applied SpaGTL to a series of multi-slice *Drosophila* Stereo-seq datasets for reconstructing three-dimensional (3D) regulatory architecture. These datasets are obtained at five developmental stages, including embryonic (E14 and E16) and larval (L1, L2, and L3) stages and preprocessed by the data provider for merging bins, slice alignment (along z axis) and clustering annotations [28] (Fig. 5a and Supplementary Figs S12–S14). We applied SpaGTL and other GRN inference models to each dataset, assessing their predictive performance using metrics such as AUROC for entire networks and Moran’s I statistic and log_2_FC for regulatory sub-networks (see Methods).

Across the five datasets, SpaGTL substantially outperformed other involved methods in both perspectives (Wilcoxon signed-rank test, $P=0.002$ for AUROC, $P<{10}^{-30}$ for mean Moran’s I and log_2_FC; Fig. 5b, c). It was also noticed that in these data, only a limited fraction of regulons (about 18% ~ 25%) was commonly identified by all tested methods (Supplementary Figs S15–S17). To further validate the regulons, we used *in situ* hybridization (ISH) data at comparable stages in embryos from Berkeley *Drosophila* Genome Project (BDGP) database [59–61]. We projected all the sections of a sample along the z-axis into a 2D graph and compared the spatial patterns of regulon activities with the TF ISH images. At different stages, the regulons refined by SpaGTL exhibit spatial active patterns that were more consistent with the shapes of ISH staining and the functional specific regions [62, 63] compared to those identified by other methods (Fig. 5d, Supplementary Figs S15, S16, andS18). Furthermore, several regulons detected exclusively by SpaGTL have been experimentally validated as region-specific. For example, SpaGTL identified the ss(+) and so(+) regulons, which are specifically active in muscle regions [59], also aligning closely with the TF ISH patterns and regulating target genes related to muscle activity or development [64] (Supplementary Fig. S19).

Additionally, we fine-tuned SpaGTL on a transverse sub-section of L3 testes to investigate putative regulons driving spatiotemporal dynamics. This tissue included continuously differentiating germ cells: spermatogonia (G), early primary spermatocytes (EPS), and late primary spermatocytes (LPS). The differentiation process is G - > EPS - > LPS, and the temporal ordering of spots is determined by RNA velocity analysis [65] (Fig. 5e and Supplementary Fig. S20). Along this pseudotime, the regulons detected by SpaGTL reflect activity dynamics that may play critical roles in state transitions (Fig. 5f, g). For instance, aop(+), serving as an inhibitor in germ differentiation (Spearman correlation = −0.76 with pseudo time order), is reported to be involved in meiosis [66] and appears as a key factor during the G and EPS stages. Egg(+) promotes the spermatogenesis (Spearman correlation = 0.78) by mediating the trimethylation of histone H3 at lysine 9 (H3K9me3) [67, 68] that controls gene expression, maintaining the cells’ differentiated state and ensuring proper differentiation progression. In brief, all evidence confirms that SpaGTL is adept at resolving 3D spatiotemporal regulatory architecture and detecting critical regulators for cell state dynamics.

## Discussion

We have developed SpaGTL, a scalable SRT regulatory analytical framework based on a novel large-scale SpaGT model, which comprises approximately 100 million parameters and is pretrained on about 100 million cells/spots to facilitate spatial context-aware GRN predictions with limited data. SpaGTL effectively captures complex gene relations from annotation-free SRT datasets and addresses the impact of regulatory networks on spatial cellular architecture. Specifically, it identifies cell types and functional regions characterized by heterogeneous regulatory programs, thereby deepening our understanding of tissue microenvironment variations and biological processes from a dynamic regulatory perspective.

To achieve this, SpaGTL incorporates specific blocks and strategies. The core model employs a gene-level graph transforms to simulate gene regulatory characteristics and a cell/spot-level manifold-dominated VAE to capture spatial graph representation. The “deep spatial distribution alignment” strategy allows SpaGTL to optimally transfer information between gene graph and cell/spot graph representation in a self-supervised way, which, on this basis, facilitates biological correspondence between identified patterns in two dimensions (i.e. gene GRNs and cell types or functional domains). Additionally, we have amassed a substantial collection of spatial resolved transcriptomics and known gene networks to pretrain the SpaGTL model, providing a robust network foundation that is pivotal for fine-tuning with limited data. SpaGTL also refines the network regulators through cis-regulatory sequence analysis, further enhancing the accuracy of deciphering spatially regulatory heterogeneity underlying tissue microenvironments.

Upon fine-tuning, the advantages of SpaGTL were validated using SRT data from various conditions. Initially, we tested the performance of SpaGTL on SRT-simulated datasets, demonstrating its superiority over existing state-of-the-art GRN inference methods in terms of precision, robustness, and speed. When applied to a 10x Visium mouse brain dataset, SpaGTL delineated finer brain structures more consistent with the reference than those methods designed specifically for spatial domain detection through expression differences. SpaGTL also uncovered the regulatory modules corresponding to these finer structures, such as Irx2(+) and Gbx2(+), distinguishing the functional heterogeneity between two spatially adjacent thalamic subregions. In applications to high-resolution datasets, SpaGTL exhibited high sensitivity to spatial context and transcriptional variation, restoring better correspondence between spatial cell types and complex molecular regulations in low-quality measurements. In Slide-seqV2 mouse cerebellum data, SpaGTL detected spatially colocalized cell types (i.e. Bergmann and Purkinje) and their key regulatory modules underlying the cell-type context. In Stereo-seq data, SpaGTL mapped the molecular regulatory landscape on 3D tissue coordinates, exploring significant regulatory modules driving cell differentiation from spermatogonia to primary spermatocytes, providing a comprehensive analysis of regulatory networks across 3D spatiotemporal scales.

In summary, SpaGTL not only enhances the analysis of gene regulatory relationships across various spatial domains and cell types but also aids in discovering regulatory modules critical in cell state transitions. This capability is essential for dissecting spatial heterogeneity in diseases and detailing the spatiotemporal blueprint of biological development. However, SpaGTL currently focuses primarily on transcriptomic data, which leaves significant room for improvement. With the continuous growth of data and the advent of advanced technologies, the data foundation for this large model will evolve further, thereby enhancing the model’s accuracy. Future expansions will also enable the incorporation of multi-omics data to more comprehensively map gene regulation and cellular behaviors. Additionally, the GRNs inferred by SpaGTL must be filtered using the cisTarget function to ensure their biological relevance. Currently, cisTarget provides regulatory knowledge specifically for *Homo sapiens*, *Drosophila melanogaster*, and *Mus musculus*, which presents a limitation for SpaGTL’s application to species beyond this scope. To address this, we plan to extend SpaGTL’s generalizability by incorporating ATAC-seq data which can directly capture the chromosome accessibility, allowing its application in scenarios with complex biological conditions or a lack of reliable knowledge.

**Key Points**

- SpaGTL is a cutting-edge graph transformer model for spatial transcriptomics, featuring approximately 100 million parameters and pretrained on nearly 100 million cells/spots. It captures complex biological patterns and spatial variations, enabling the discovery of key network regulators in fine-tuned or data-limited SRT datasets.
- SpaGTL represents a novel cross-dimensional transfer learning architecture that integrates tailored neural networks for gene and cell dimensions, enhanced by a contrastive encoder-decoder for self-supervised alignment. It bridges regulatory network identification and cellular ecosystem analysis, revealing dynamic regulatory and cellular interactions in tissues.
- SpaGTL supports diverse SRT platforms, multi-modality/slice integration, and external tools like “anndata”. Its user-friendly design makes it essential for studying complex biological systems with advanced computational approaches.

## Supplementary Material

SpaGTL_SI_bbaf021

## Data Availability

The DLPFC data is publicly available from the Bioconductor package spatialLIBD (Zenodo https://doi.org/10.5281/zenodo.3689719) or can be downloaded at http://research.libd.org/globus. The mouse brain coronal 10x Visium data can be downloaded from the 10x Genomics official website at https://www.10xgenomics.com/resources/datasets/. The annotation for this slice is referenced from the Allen Brain Map database: https://atlas.brain-map.org/. The Slide-seqV2 mouse cerebellar data are obtained from the Broad Institute Single Cell Portal available at https://singlecell.broadinstitute.org/single_cell/study/SCP948. The *Drosophila* embryo and larval Stereo-seq datasets are downloaded from Flysta3D database: https://db.cngb.org/stomics/flysta3d/. The reference *Drosophila* regulatory network used for GRNs inference evaluation is downloaded from http://www.droidb.org/. The *Drosophila* larval ISH sections are obtained from BDGP database: https://insitu.fruitfly.org/. Python source code of SpaGTL, under the open-source BSD 3-Clause license, is available at https://github.com/zccqq/SpaGTL. The documentation website provides the installation guide, tutorials, and API references, which is available at https://spagtl.readthedocs.io/. SpaGTL is also published as a Python package named “spagtl” on Python Package Index (PyPI) at https://pypi.org/project/ spagtl/ and can be directly installed via the pip installer.
