# Supplementary material for: Spatially aligned graph transfer learning for characterizing spatial regulatory heterogeneity
Source: Brief Bioinform. 2025 Jan 22;26(1):bbaf021. doi: 10.1093/bib/bbaf021 (PMC11752617; doi:10.1093/bib/bbaf021)
Supplement: SpaGTL_SI_bbaf021 [file spagtl_si_bbaf021.pdf]

Spatially aligned graph transfer learning for characterizing spatial regulatory heterogeneity

Wendong Huang[1,2], Yaofeng Hu[3], Lequn Wang[4], Guangsheng Wu[5], Chuanchao Zhang[3*], Qianqian Shi[1,2*]

[1]Hubei Key Laboratory of Agricultural Bioinformatics, College of Informatics, Huazhong Agricultural University, Wuhan 430070, China

[2]Hubei Engineering Technology Research Center of Agricultural Big Data, Huazhong Agricultural University, Wuhan 430070, Hubei, China

[3]Key Laboratory of Systems Health Science of Zhejiang Province, School of Life Science, Hangzhou Institute for Advanced Study, University of Chinese Academy of Sciences, Hangzhou, 310024, China

[4]State Key Laboratory of Cell Biology, Shanghai Institute of Biochemistry and Cell Biology, Center for Excellence in Molecular Cell Science, Chinese Academy of Sciences, Shanghai 200031, China

[5]School of Mathematics and Computer Science, Xinyu University, Xinyu 338004, Jiangxi, China

* To whom correspondence should be addressed. Qianqian Shi. Email: qqshi@mail.hzau.edu.cn; Chuanchao Zhang, Email: chuanchaozhang@ucas.ac.cn

Section S1. Supplementary Notes:

1.1 Simulation data generation

To evaluate GRNs inference performance in SRT data, we created several simulated datasets. Each dataset includes 1) the gene network that serves as the ground truth for performance evaluation, 2) the expression measurements and locational information, both of which are contained in a SRT dataset.

First, we used BoolODE [1] to generate the cellular-level expression datasets. BoolODE is a single-cell transcriptomic data simulation method, which can convert Boolean functions specifying a prior network directly to ODE equations. Based on dynamic differential equations, the BoolODE expressions are inferred underlying the input network that in turn can be regarded as the ground truth for GRNs inference evaluation. In this work, we used two sets of regulatory networks for simulation. One comprises regulatory networks with biological priors, which were integrated from three published regulatory network models, namely Mammalian Cortical Area Development (mCAD) [2], Hierarchical differentiation of myeloid (HSC) [3], and Gonadal Sex Determination (GSD) [4]. The combined network contains 35 TFs and 123 interactions (Supplementary Fig. 1a). In the other, we use 'toy' networks created in Dynverse [5], contains 21 TFs and 40 interactions (Supplementary Fig. 2a). Thus, we generated the single cell synthetic datasets respectively named as data1 and data2 based on the two regulatory networks. For simplicity, each dataset contains 10,000 cells.

Then, with the simulated datasets, we performed *t*-SNE dimensionality reduction (Supplementary Fig. 1b and 2b). We regarded the *t*-SNE embeddings of cells, i.e., x-y coordinates, as the "pseudo" spatial locations. Based on the locations, we divided the cells into equal-sized bins, and generated the positional expression profiles of each bin by adding the expression values of all the merged cells [6]. After merging, the new expression values with refined coordinates therefore could be a simulated SRT dataset. As we know that the mainstream SRT technologies differ in the resolved resolutions, e.g., 8 ~ 10 cells per spot for 10x Visium and 1 ~ 3 cells for Slide-seqV2, the bin size is a critical factor in simulation. According to 10x Visium and Slide-seqV2 resolution levels, we tuned the bin size and finally obtained four sets of simulated data (with 972 ~ 5,038 spots) underlying the settled networks (Supplementary Fig. 1 and 2).

1.2 Comparison of other spatial domain identification methods

We adopt the same preprocessing procedure as SpaGTL for other methods. We implement these methods according to their tutorial websites or code scripts posed on GitHub repository. We implement each method using the default parameters or parameters specified in their tutorials or code scripts with the only exception of adjusting the number of clusters or clustering resolution to guarantee that each method identifies the same number of spatial domains for fair comparison.

The reference implementation of tutorial website or code script for each method is listed below:

- STAGATE: https://stagate.readthedocs.io/en/latest/T1_DLPFC.html
- DeepST: https://github.com/JiangBioLab/DeepST

- GraphST: https://deepst-tutorials.readthedocs.io/en/latest/Tutorial%201_10X%20Visium.html
- SpaGCN: https://github.com/jianhuupenn/SpaGCN/blob/master/tutorial/tutorial.md

We utilize the newest version (until October 2023) of each method to accommodate the new features and improvements. The version of each method is listed below: STAGATE v1.0.1; DeepST code scripts on GitHub; GraphST v1.1.1; SpaGCN v1.2.7.

1.3 Comparison with other GRNs inference methods

To evaluate the performance of GRNs inference, we compared SpaGTL with GRNboost2, Genie3, PIDC, DeepSEM, DGRNs scSGL, SpaceX and Hotspot.

- GRNboost2: We followed the workflow specified in the example of arboreto documentation website (https://arboreto.readthedocs.io/en/latest/examples.html). Firstly, we downloaded a list of transcription factors for the studied species from the cistarget database (https://resources.aertslab.org/cistarget/tf_lists/). Then, we inferred GRNs using the counts expression matrix and transcription factor list as inputs to function grnboost2(). During the process of running grnboost2(), all parameters used default parameters.
- Genie3: We followed the workflow specified in the example of arboreto documentation website (https://arboreto.readthedocs.io/en/latest/examples.html). Firstly, we downloaded a list of transcription factors for the studied species from the cistarget database (https://resources.aertslab.org/cistarget/tf_lists/). Then, we inferred GRNs using the counts expression matrix and transcription factor list as inputs to function genie3(). During the process of running genie3(), all parameters used default parameters.
- PIDC: We followed the workflow specified in the example of NetworkInference.jl documentation website (https://github.com/Tchanders/NetworkInference.jl). Firstly, we used the counts expression matrix as input to the get_nodes() function to obtain an array of network nodes. Then, we inferred GRNs using array of network nodes as inputs to function InferredNetwork(). During the process of running InferredNetwork(), We specified the value of the first parameter of the function as PIDCNetworkInference().
- DeepSEM: We followed the workflow specified in the example of DeepSEM documentation website (https://github.com/HantaoShu/DeepSEM). we used the counts expression matrix as input, and set the 'n epochs' parameter to 120, 'alpha' parameter to 1, 'beta' parameter to 0.01, following the tutorial recommendation.
- DGRNs: We followed the workflow specified in the example of DGRNs documentation website (https://github.com/MengyuanZhaoo/DGRNS). Firstly, we run gen_genePairList.py to generate gene pair list file from gene expression file and gold network. In the real dataset, we used the gold network provided by BEELINE (https://zenodo.org/records/3701939) as an input required by the model. Secondly, we run gen_fullmatrix_TF.py to generate correlation vectors from gene pair list and gene

expression profiles. Thirdly, we run gen_datasetSplit.py to split dataset into training set, validation set and test set. Next, we run DGRNS_train.py to train DGRNS, and run predict_fullnetwork.py to construct gene regulation relationships. During the process of running, all parameters used default parameters.

- scSGL: We followed the workflow specified in the example of scSGL documentation website (https://github.com/Single-Cell-Graph-Learning/scSGL/blob/main/notebooks/demo.ipynb). We set the 'pos_density' parameter to 0.45, 'neg_density' parameter to 0.45, 'assoc' parameter to correlation, following the tutorial recommendation.
- SpaceX: We followed the workflow specified in the example of SpaceX documentation website (https://github.com/bayesrx/SpaceX). The SpaceX() requires prior clustering information. Therefore, we performed pre-clustering on the dataset using Seurat with default parameters.
- Hotspot: We followed the workflow specified in the example of Hotspot documentation website (https://hotspot.readthedocs.io/en/latest/Spatial_Tutorial.html). We employed the hotspot.Hotspot() to initialize the Hotspot object, subsequently constructed the neighborhood graph using hotspot.create_knn_graph(), and ultimately computed the correlation matrix across all genes by invoking hs.compute_local_correlations().

1.4 Fine-tuning SpaGTL on limited SRT data

Fine-tuning was accomplished by initializing the model with the pretrained parameter library and retraining the core model for capturing the regulation structures and spot/cell-level graph representation of limited SRT data. Due to the limited amount of fine-tuning data for SRT, we compress gene-level transfer learning into a single transformer encoder unit that only contains a full dense self-attention layer. This simplification allows the model to utilize the fine-tuning dataset effectively. Additionally, we use the pretrained attention weight parameters S as the initial values for the fine-tuning attention weights. The initial values of other parameters are extracted from the pretrained parameter library based on the genes involved in the computations.

Section S2. Supplementary Figures and Tables

Supplementary Figure 1: Sensitivity analysis of the parameter μ. The parameter μ (i.e., "Mu") is used to ensure that gene-gene relations with realistic record can obtain higher attention weight value and achieves the biological significance of the inferred GRNs in the pretraining phase. To test the effect of the parameter μ on model performance, we pretrained SpaGTL on large-scale SRT datasets while varying the parameter μ from 0 to 1 (20 repeats for each parameter). After pretraining, we calculated the AUC value to quantify prediction accuracy of the learned weights S compared to the ground-truth network A **(a)** and computed the ARI value of clustering results on 12 the dorsolateral prefrontal cortex (DLPFC) with annotation information [7] **(b)**. From the results, it can be seen that the continuous increase of parameter μ improves the biological meaning of inferred GRNs while reducing the discriminative power (i.e., spatial domains detection) of the model in terms of tissue heterogeneity. Therefore, the reasonable value range of parameter μ should be 0.1~0.3, and the default value in this paper is 0.2.

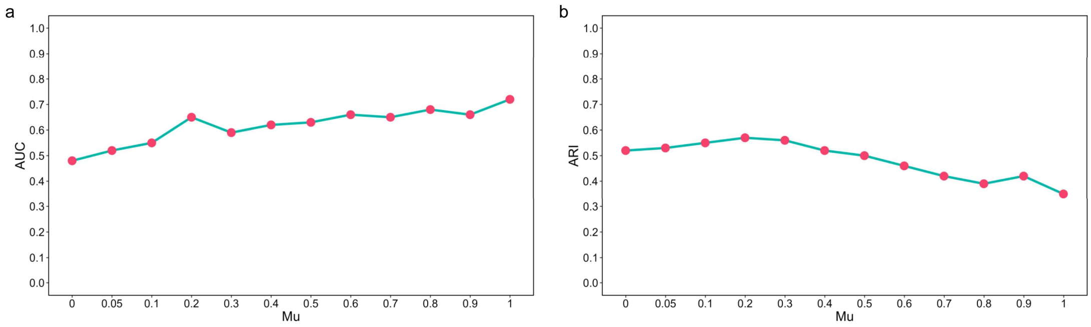

Supplementary Figure 2: SpaGTL uses the alpha parameter to flexibly incorporate spatial information into spatial domain identification and regulatory networks inference on the IDC sample (*n* = 4,727 spots). (**a**) Spatial domains generated by SpaGTL under a variety of alpha settings. Alpha controls the extent to which gene expression borrows information from neighboring spots, while a larger alpha will aggregate more information from the spatial neighborhood. The identified spatial domains are distinguished using different colors and are shown *in situ* against the immunofluorescent image. The cluster purity is used to compare the similarity between the identified spatial domains and the ground truth annotations provided in the original publication [8]. (**b**) The spatial distribution of representative regulons (i.e., ELK3(+)) identified by SpaGTL under a variety of alpha settings. (**c**) Spatial coherence measurements of the identified domains from (**a**). Moran's I measures the domain-wise global spatial autocorrelation (for 10 domains). (**d**) Spatial coherence measurements of the inferred regulons via Moran's I values.

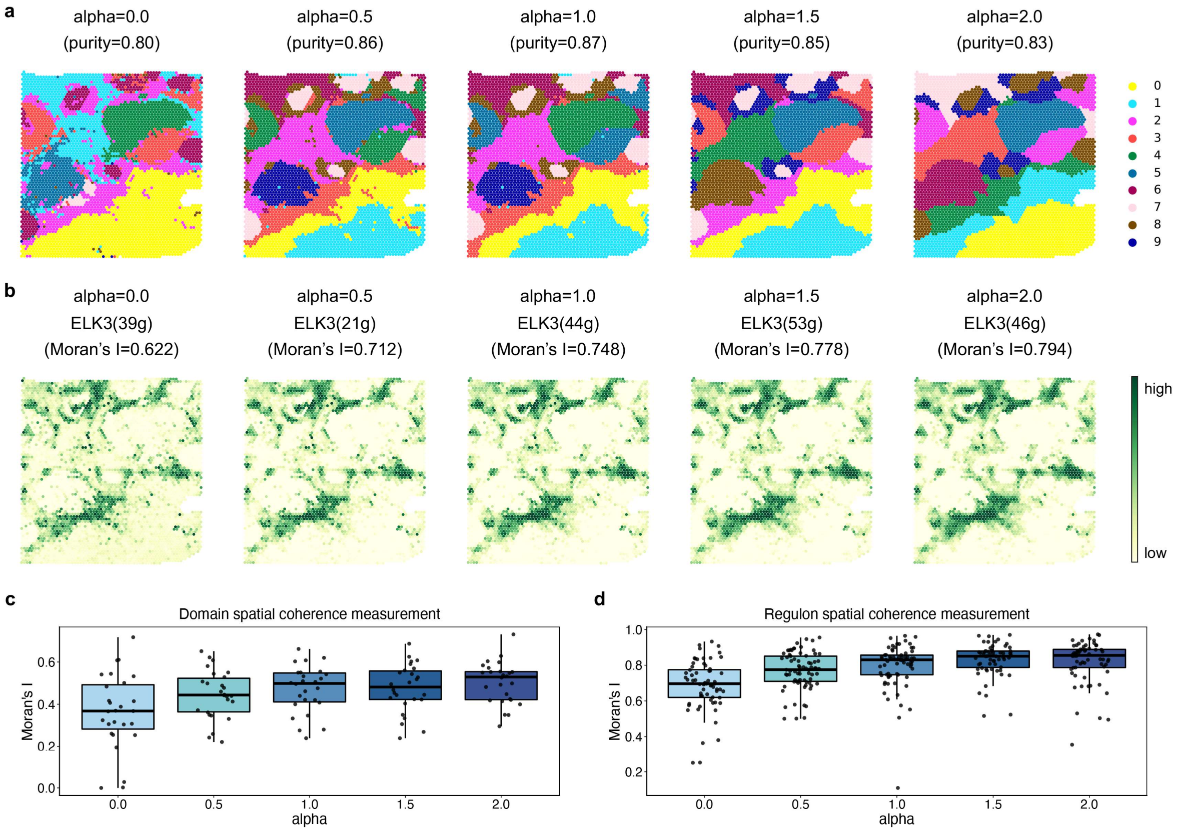

Supplementary Figure 3: Sensitivity analysis of the parameters $\boldsymbol{\lambda_1}$ and $\boldsymbol{\lambda_2}$. The parameters λ_1 and λ_2 (i.e., "Lambda_1" and "Lambda_2") control the spot-level graph representation learning. To test the effect of the parameters λ_1 and λ_2 on model performance, we pretrained SpaGTL on large-scale SRT datasets while varying the parameters λ_1 and λ_2 from 0 to 5 ($\times$ 10000) and 0 to 9 ($\times$ 10000) (20 repeats for each parameter), respectively. After pretraining, we calculated the ARI values of clustering results on 12 the dorsolateral prefrontal cortex (DLPFC) with annotation information [7]. SpaGTL performs robust within a certain range (λ_1: 0.2~2 ($\times$ 10000); λ_2: 1~4 ($\times$ 10000)). Since parameter λ_2 controls the contrast component (i.e., $\mathcal{L}_{CL}$), parameter λ_2 requires bigger values than parameter λ_1 to effectively improve the model performance.

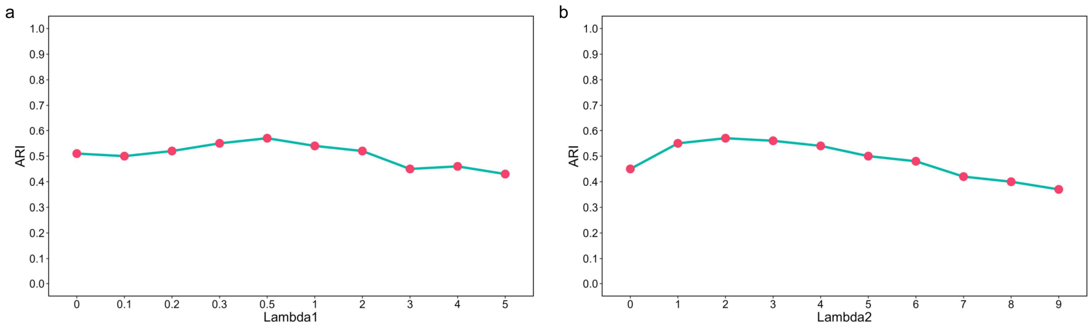

Supplementary Figure 4: Ablation experiments of SpaGTL. We systematically evaluated the contribution of different components in the pretrained SpaGTL model to its overall performance. We sequentially removed these components from the model and calculated AUC and ARI values on 12 DLPFC. We observed that "SpaGTL(-align_GD)" and "SpaGTL(-align_SLD)", which excluded global and spatially local distribution alignment component (i.e., $\mathcal{L}_{align_{GD}}$ and $\mathcal{L}_{align_{SLD}}$) and , achieved higher ARI values, and "SpaGTL(-align_CL)", which excluded contrast component (i.e., $\mathcal{L}_{CL}$), obtained the lowest ARI values. However, the ARI values for these modified models were still lower than that of the complete SpaGTL model. This suggests that all components provide valuable information to the model, but contrast component are particularly important. Contrast component not only provide constraints of the unified graph representation learning but also play a crucial role in the implementation of deep spatial distribution alignment strategy. Furthermore, we evaluated the performance of the unified graph representation learning strategy by running stMDA without deep self-expressive component (i.e., "SpaGTL(-align_manifold)") and without the contrast component (i.e., "SpaGTL(-align_CL)"). We found that the contrast component still significantly constrains the performance of SpaGTL during the unified graph representation learning.

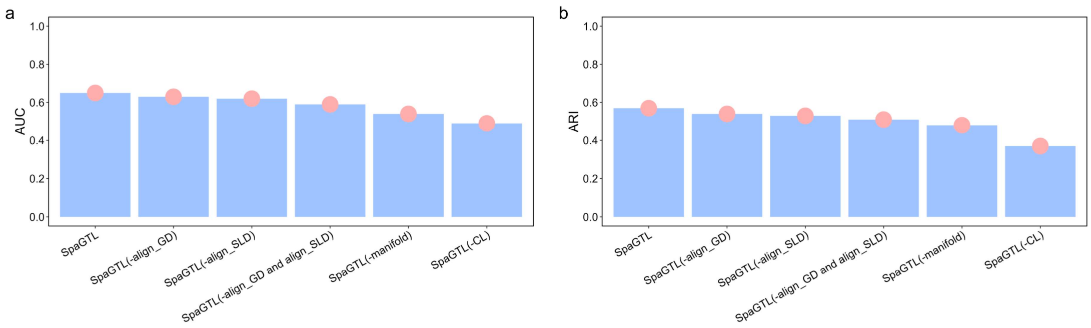

Supplementary Fig. 5: Generate simulated SRT data1 from known gene regulatory relationships. (a) The true regulatory network used as input for the BoolODE model. Each point represents a gene, and the arrows indicate the direction of regulation. The network consists of a total of 35 nodes and 123 edges. **(b)** Single-cell level expression data generated by BoolODE. The data comprises 10,000 cells, reduced using *t*-SNE, with the color of each cell representing the total expression value of the cell. **(c)** The result of binning for (b), with each bin considered as a 'spot.' The horizontal axis represents the number of cells contained in each spot, and the vertical axis represents the number of spots. Many spots contain between 8-10 cells, which is close to the resolution of 10x Visium. **(d)** The expression values of spots in spatial coordinates, obtained from *t*-SNE coordinates. After merging, there are a total of 972 spots. **(e)** The result of binning for (b). Most spots contain between 1-3 cells, which is close to the resolution of Slide-seqV2. **(f)** The expression values of spots in spatial coordinates, obtained from *t*-SNE coordinates. After merging, there are a total of 5038 spots.

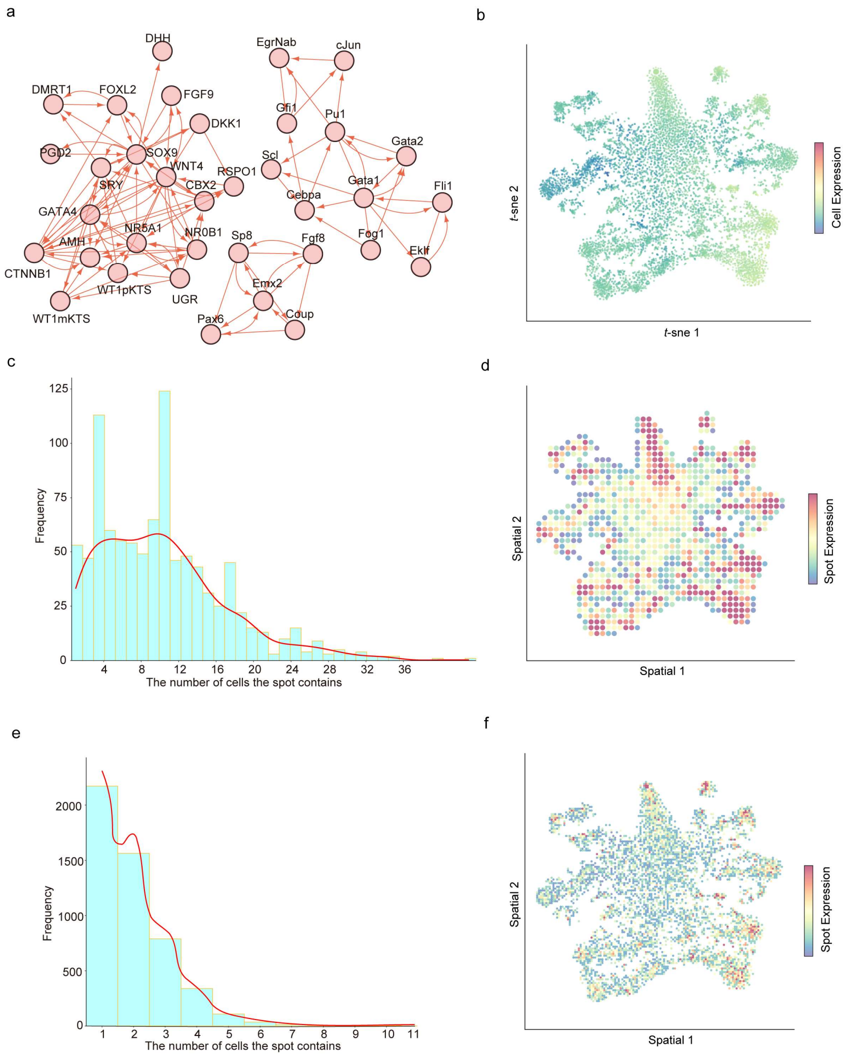

Supplementary Fig. 6: Generate simulated SRT data2 from toy gene regulatory relationships. (a) The toy regulatory network used as input for the BoolODE model. The network consists of a total of 21 nodes and 40 edges. **(b)** Single-cell level expression data generated by BoolODE, the data comprises 10,000 cells. **(c)** The result of binning for (b). Many spots contain between 8-10 cells, which is close to the resolution of 10x Visium. **(d)** The expression values of spots in spatial coordinates, there are a total of 1182 spots. **(e)** The result of binning for (b). Most spots contain between 1-3 cells, which is close to the resolution of Slide-seqV2. **(f)** The expression values of spots in spatial coordinates, there are a total of 4755 spots.

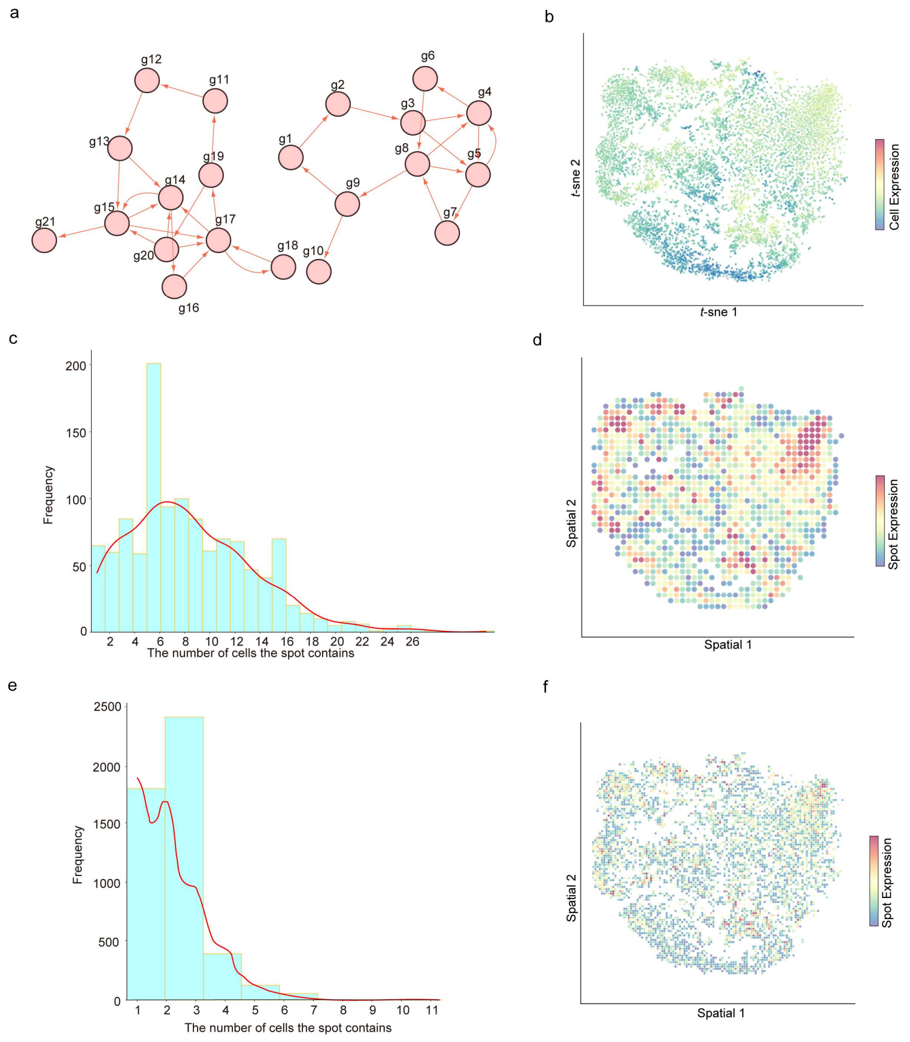

Supplementary Fig. 7: The clustering performance of SpaGTL on DPLEC data. (a) Comparison of clustering accuracy. Clustering was performed using 1,000, 2,000, 3,000, 4,000, and 5,000 highly variable genes, and the Adjusted Rand Index (ARI) was calculated for each scenario. Groups were compared using a Wilcoxon rank sum test with significance levels marked as * $p < 0.05$. **(b)** Runtime for Clustering Using 1,000, 2,000, 3,000, 4,000, and 5,000 highly variable genes. **(c)** Spatial visualization of clustering results using 4,000 highly variable genes.

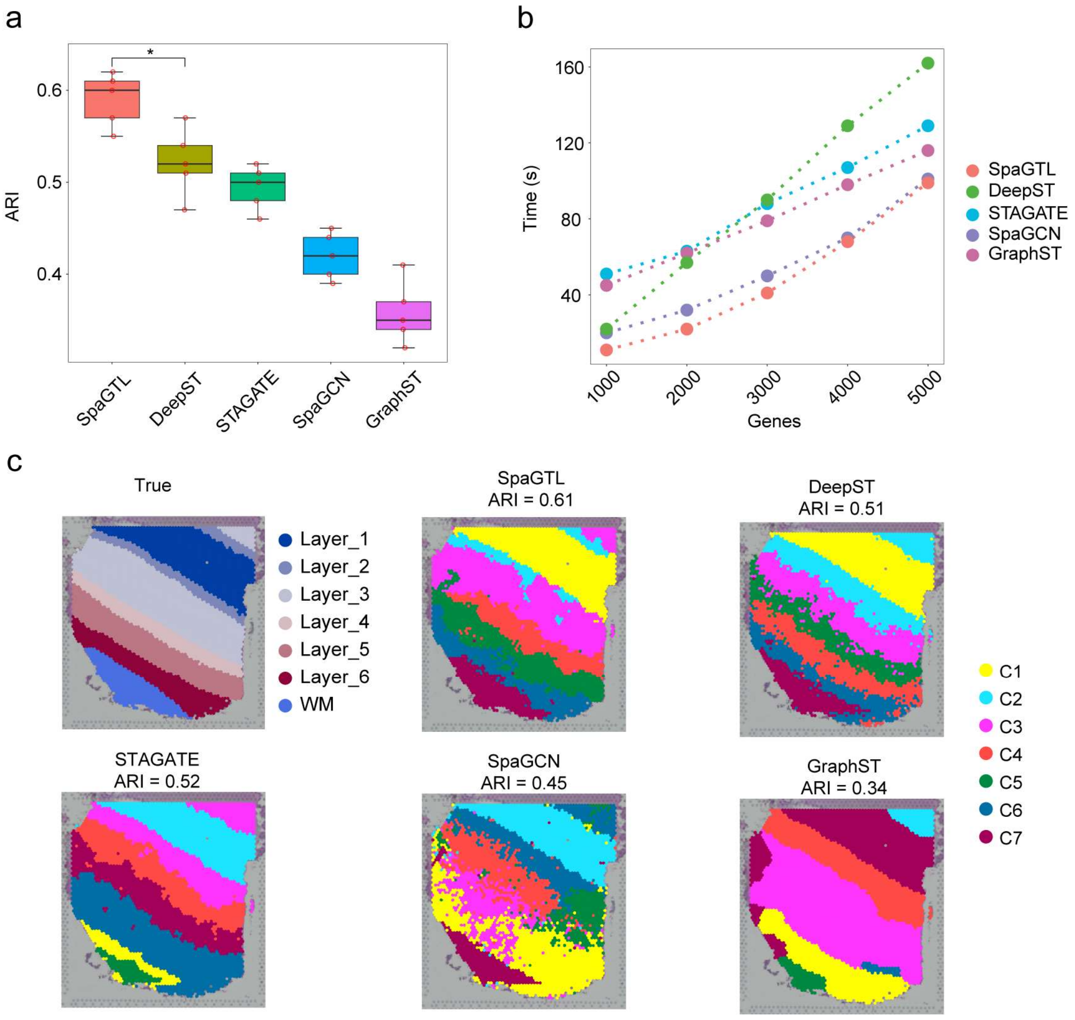

Supplementary Fig. 8: The clustering performance of SpaGTL on 10x Visium data. (a) Comparison with other clustering methods. The left panel shows the annotation information from the Allen Brain Atlas, and the right panel presents the clustering results of various methods. **(b)** Heatmap of differentially expressed genes in the clustering results of SpaGTL. **(c)** Spatial patterns of differential genes expression for each cluster. The top panel shows the spatial distribution of clusters, the middle panel illustrates the spatial expression of differential genes, and the bottom panel displays the ISH information of differential genes (sourced from the Allen Brain Atlas).

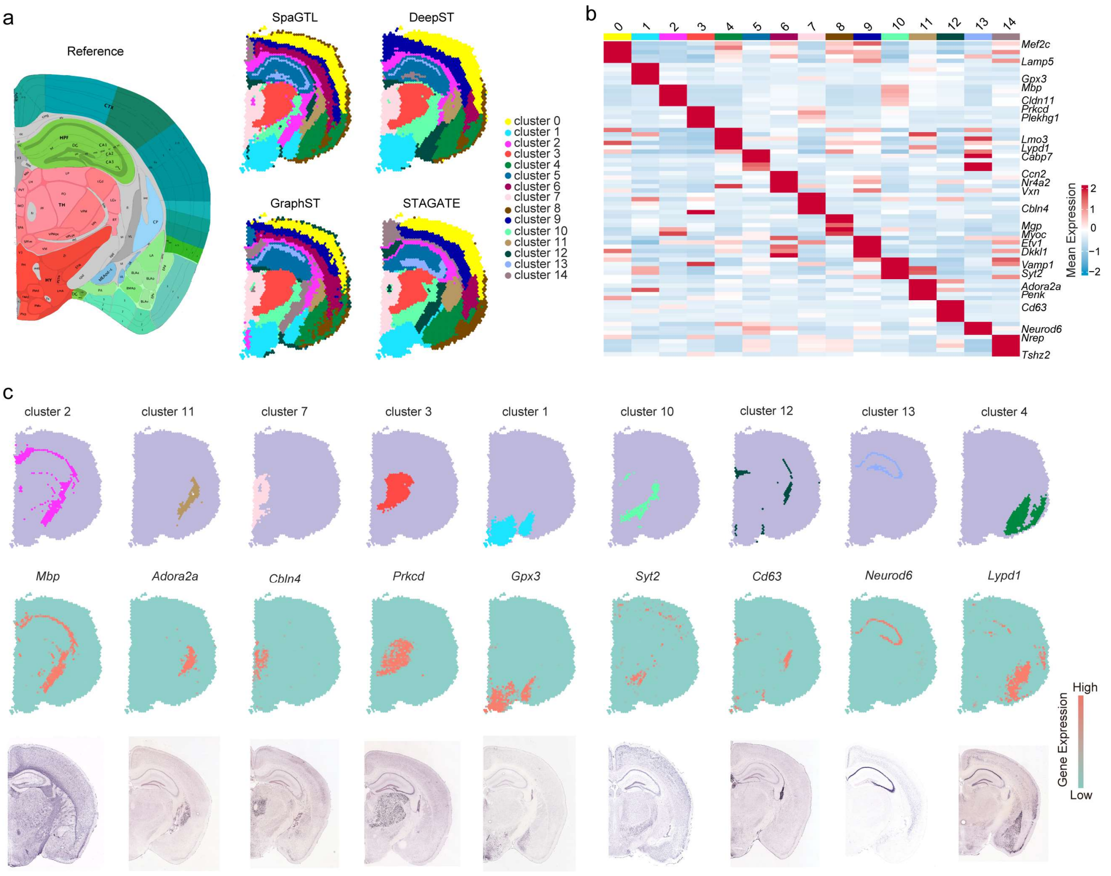

Supplementary Fig. 9: Spatial regulatory patterns of selected marker regulons of different domains. The corresponding domains are shown on the left; TFs expression is displayed in the second column. The right panel presents the spatial patterns of regulons activity.

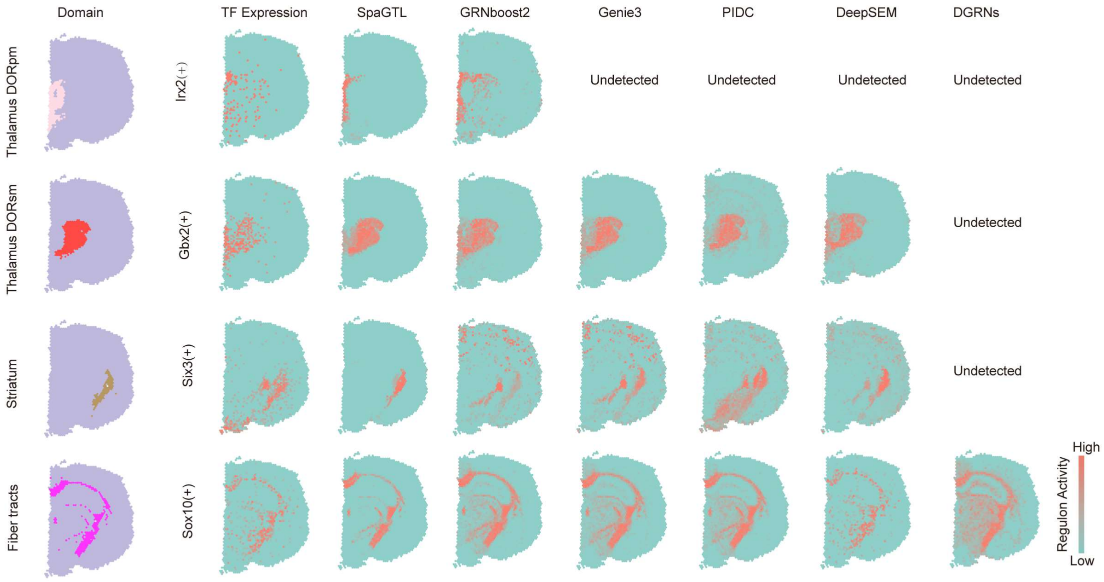

Supplementary Fig. 10: The clustering performance of SpaGTL on Slide-seqV2 data. (a) Comparison with other clustering methods. **(b)** Heatmap of marker genes in the clustering results of SpaGTL. **(c)** Spatial patterns of marker gene expression for each cluster. The top panel shows the spatial distribution of clusters, the middle panel illustrates the spatial expression of marker genes, and the bottom panel displays the ISH information of marker genes (sourced from the Allen Brain Atlas).

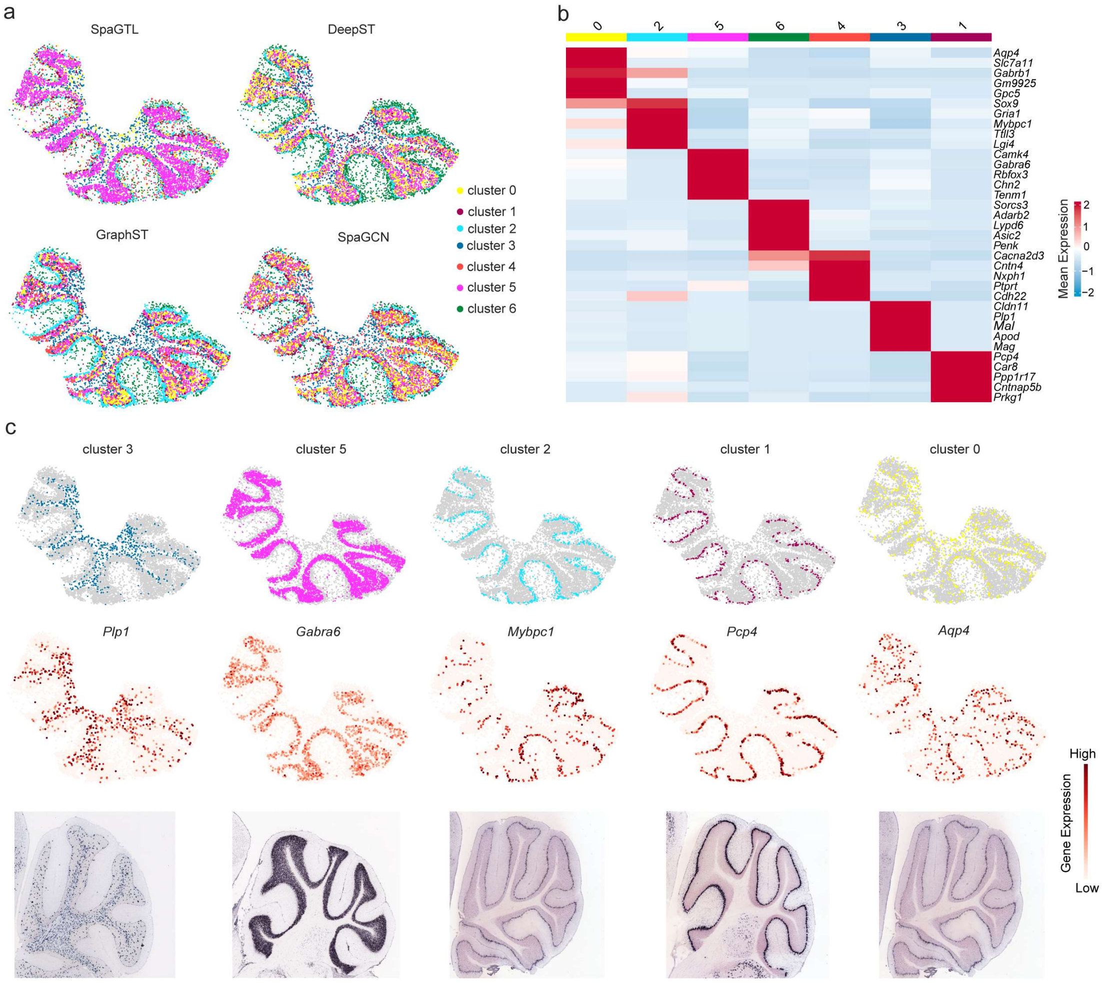

Supplementary Fig. 11: Spatial regulatory patterns of selected marker regulons of different cell types. The corresponding cell type are shown on the left; TFs expression and ISH are displayed in the second and third columns, with the ISH information sourced from the Allen Brain Atlas. The right panel presents the spatial patterns of regulons activity.

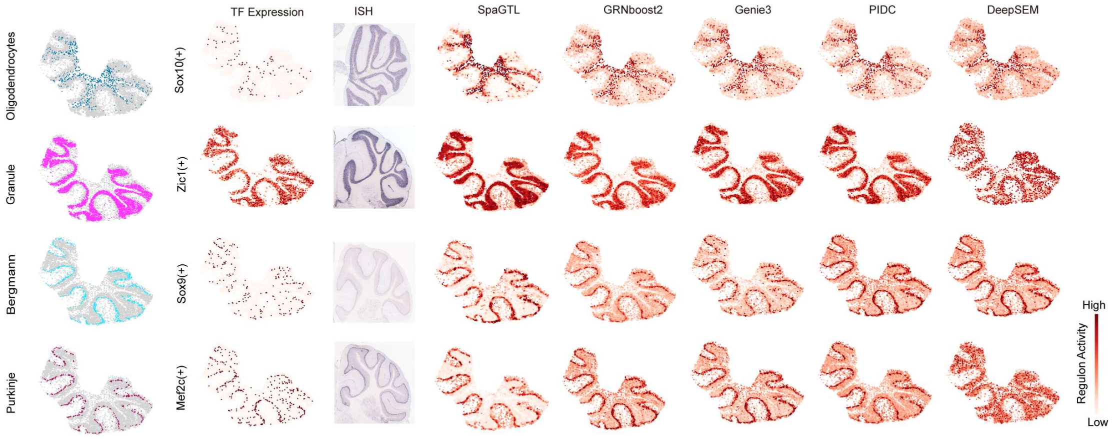

Supplementary Fig. 12: The spatial domain identification on 3D *Drosophila* embryonic E14 data. The identified spatial domains by SpaGTL, SpaGCN, STAGATE are distinguished by distinctive colors without correspondence. Cluster purity (i.e., purity) is used to evaluate the clustering performance.

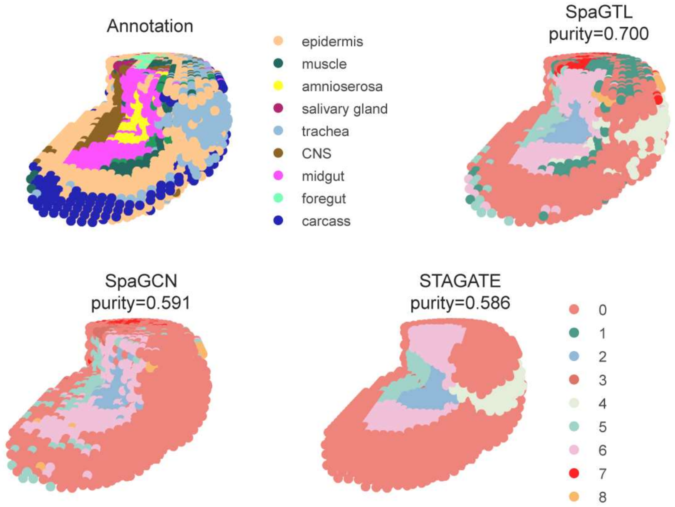

Supplementary Fig. 13: The spatial domain identification on 3D *Drosophila* embryonic E16 data. The identified spatial domains by SpaGTL, SpaGCN, STAGATE are distinguished by distinctive colors without correspondence. Cluster purity (i.e., purity) is used to evaluate the clustering performance.

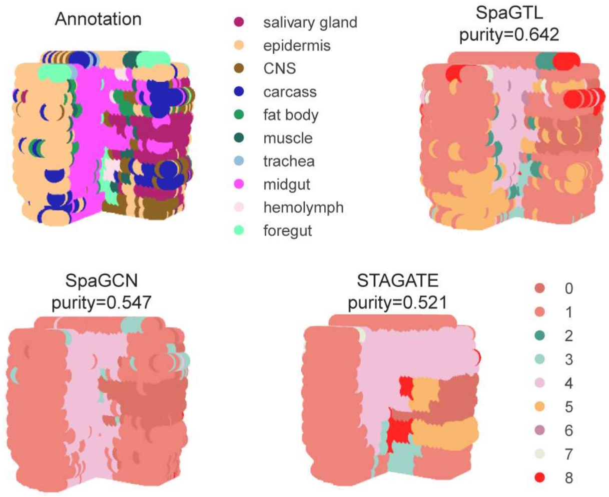

Supplementary Fig. 14: The spatial domain identification on 3D *Drosophila* embryonic L1 data. The identified spatial domains by SpaGTL, SpaGCN, STAGATE are distinguished by distinctive colors without correspondence. Cluster purity (i.e., purity) is used to evaluate the clustering performance.

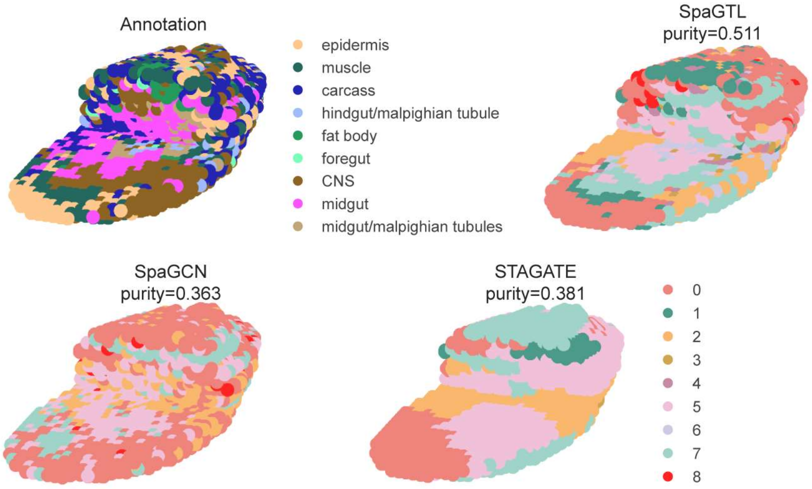

Supplementary Fig. 15: Regulons with spatial expression patterns in the E14 dataset. (a) UpSet plot of inferred sets of regulons by the five methods in the E14 dataset. 11 regulons were identified by all methods, and some of these regulons exhibit spatial patterns of regulation, such as grh(+), Rfx(+), and bs(+). **(b)** Regulons with spatial patterns found in the E14 dataset. These regulons all belong to the 11 regulons in (a). The corresponding domains are shown on the left; TFs ISH sourced from Berkeley Drosophila Genome Project (BDGP) database are displayed on the right; the middle panel presents the spatial patterns of regulons activity.

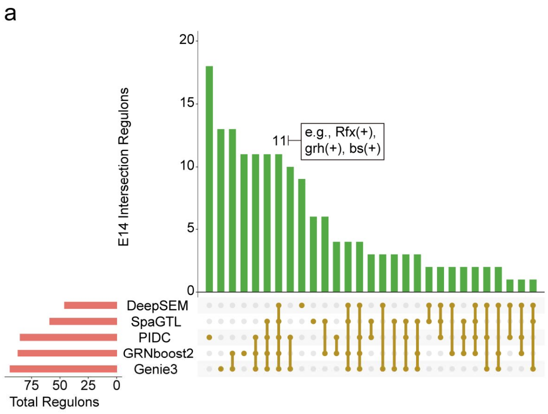

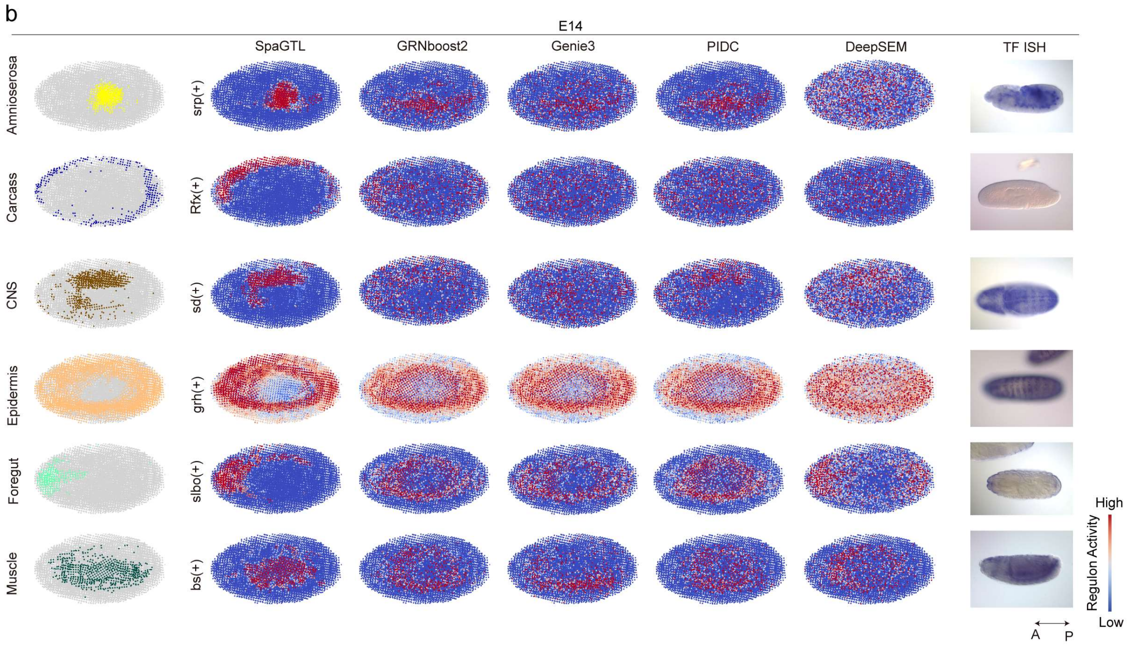

Supplementary Fig. 16: Regulons with spatial expression patterns in the L1 dataset. (a) UpSet plot of inferred sets of regulons by the five methods in the L1 dataset. 12 regulons were identified by all methods, and some of these regulons exhibit spatial patterns of regulation, such as Rbp6(+), Rx(+), and bin(+). **(b)** Regulons with spatial patterns found in the L1 dataset. These regulons all belong to the 12 regulons in (b). The corresponding domains are shown on the left; TFs ISH sourced from Berkeley Drosophila Genome Project (BDGP) database are displayed on the right ; the middle panel presents the spatial patterns of regulons activity.

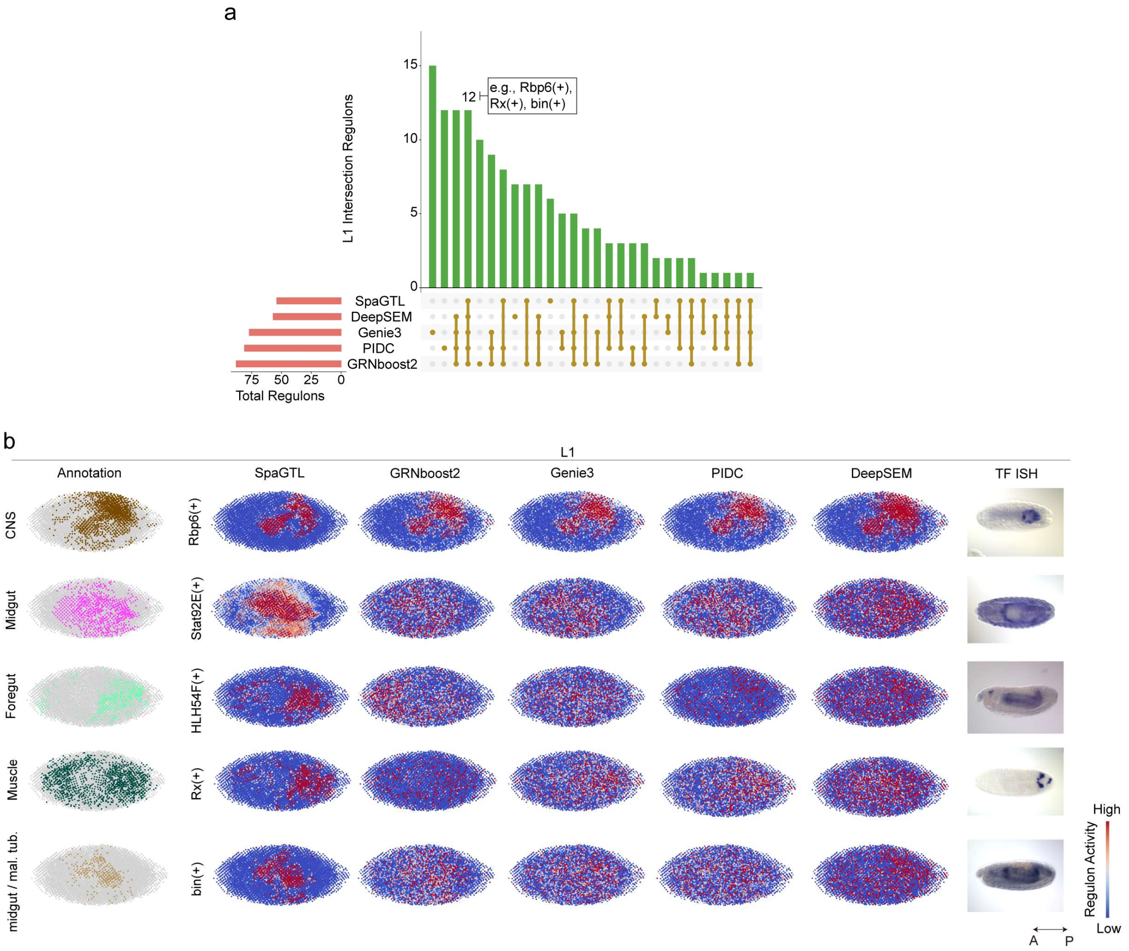

Supplementary Fig. 17: UpSet plot of inferred sets of regulons by the five methods in the E16 dataset. The bar plot at the top shows the amount of intersection results indicated by dots. The arrows highlight the representative regulons inferred by different methods. The bar plot on the left shows the total numbers of inferred regulons for each method.

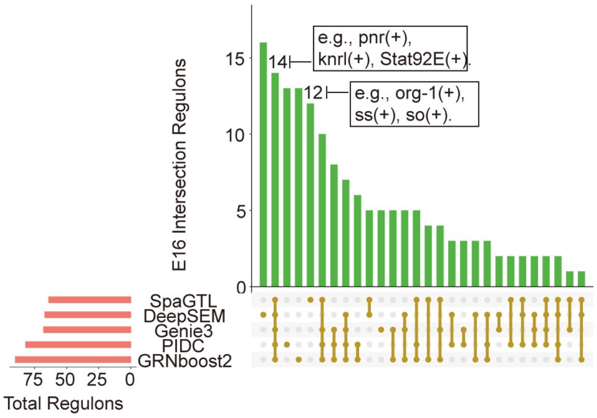

Supplementary Fig. 18: Regulons with spatial expression patterns in the E16 dataset. The corresponding domains are shown on the left; TFs ISH sourced from Berkeley Drosophila Genome Project (BDGP) database are displayed on the right; the middle panel presents the spatial patterns of regulons activity.

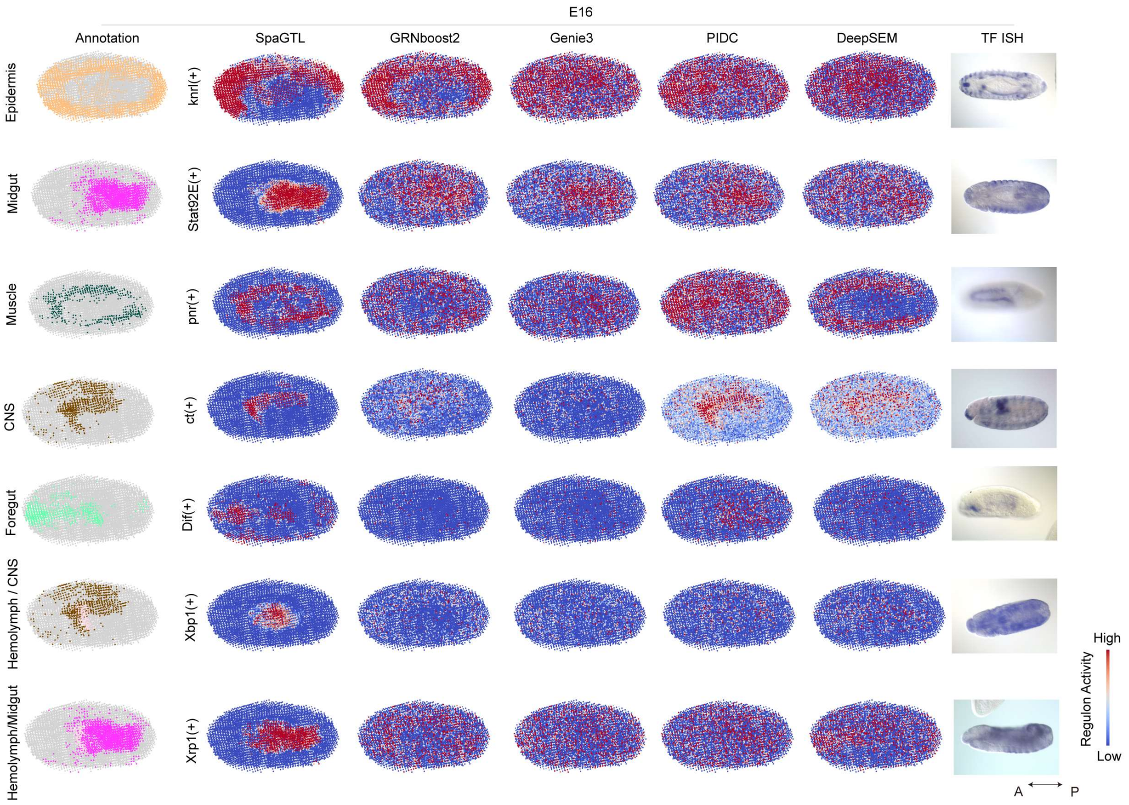

Supplementary Fig. 19: Some regulons identified exclusively by SpaGTL in the E16 dataset are presented. (a) regulons with spatial activity pattern. From left to right, the region annotation, the spatial patterns of regulons activity, the ISH images from BDGP database are respectively shown. **(b)** The network topology of ss(+) and so(+) regulons. Each node indicates a gene and each directed edge (from TF to target) indicates a putative regulation interaction. Node colors represent the involved biological functions of genes.

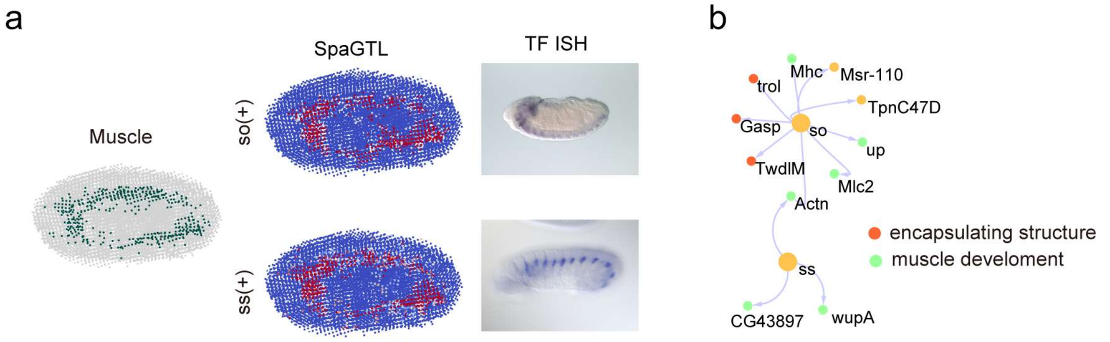

Supplementary Fig. 20: Annotation and RNA velocity analysis of the testis from L3 transverse section. After subdividing the bin 50 × 50 DNA nanoballs (DNBs) into bin 15×15 DNBs, we determined the cell types that each merged bin (bin 15×15 DNBs) represents by mapping Stereo-seq data to an annotated larval testis scRNA-seq dataset. Subsequently, E, LPS, and EPS were selected for RNA velocity analysis.

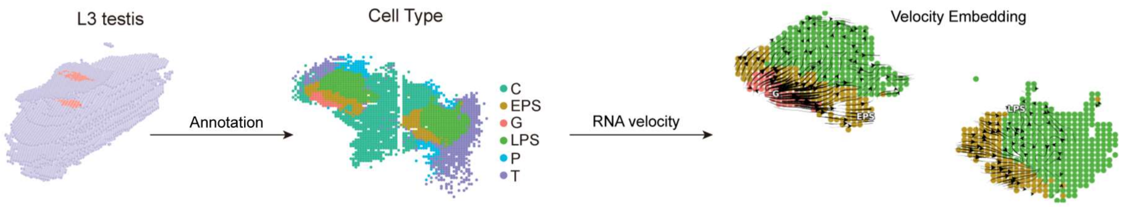

Reference

1. Pratapa A, Jalihal AP, Law JN , et al. Benchmarking algorithms for gene regulatory network inference from single-cell transcriptomic data. *Nat Methods* 2020; 17(2):147-154.
2. Giacomantonio CE, Goodhill GJ. A Boolean model of the gene regulatory network underlying Mammalian cortical area development. *PLoS Comput Biol* 2010; 6(9):e1000936.
3. Krumsiek J, Marr C, Schroeder T , et al. Hierarchical differentiation of myeloid progenitors is encoded in the transcription factor network. *PLoS ONE* 2011; 6(8):e22649.
4. Ríos O, Frias S, Rodríguez A , et al. A Boolean network model of human gonadal sex determination. *Theor Biol Med Model* 2015; 12(1):1-18.
5. Saelens W, Cannoodt R, Todorov H , et al. A comparison of single-cell trajectory inference methods. *Nat Biotechnol* 2019; 37(5):547-554.
6. Stickels RR, Murray E, Kumar P , et al. Highly sensitive spatial transcriptomics at near-cellular resolution with Slide-seqV2. *Nat Biotechnol* 2021; 39(3):313-319.
7. Maynard KR, Collado-Torres L, Weber LM , et al. Transcriptome-scale spatial gene expression in the human dorsolateral prefrontal cortex. *Nat Neurosci* 2021; 24(3):425-436.
8. Zhao E, Stone MR, Ren X , et al. Spatial transcriptomics at subspot resolution with BayesSpace. *Nat Biotechnol* 2021; 39(11):1375-1384.
